# Local delivery of hrBMP4 as an anticancer therapy in patients with recurrent glioblastoma: a first-in-human phase 1 dose escalation trial

**DOI:** 10.1186/s12943-023-01835-6

**Published:** 2023-08-10

**Authors:** Eelke M. Bos, Elena Binda, Iris S.C. Verploegh, Eva Wembacher, Daphna Hoefnagel, Rutger K. Balvers, Anne L. Korporaal, Andrea Conidi, Esther A. H. Warnert, Nadia Trivieri, Alberto Visioli, Paola Zaccarini, Laura Caiola, Rogier van Wijck, Peter van der Spek, Danny Huylebroeck, Sieger Leenstra, Martine L.M. Lamfers, Zvi Ram, Manfred Westphal, David Noske, Federico Legnani, Francesco DiMeco, Angelo Luigi Vescovi, Clemens M.F. Dirven

**Affiliations:** 1 Department of Neurosurgery, Erasmus MC Cancer Institute, Erasmus University Medical Center, Rotterdam, The Netherlands; 2 Unit of Cancer Stem Cells, ISBReMIT, IRCCS CasaSollievo della Sofferenza, San Giovanni Rotondo (FG), Italy; 3 Department of Cell Biology, Erasmus MC Cancer Institute, Erasmus University Medical Center, Rotterdam, The Netherlands; 4 Brainlab A.G., Munich, Germany; 5 Department of Radiology, Erasmus MC Cancer Institute, Erasmus University Medical Center, Rotterdam, The Netherlands; 6 StemGen SpA, Milan, Italy; 7 HyperStem SA, Lugano, Switzerland; 8 Department of Clinical Bioinformatics, Erasmus MC Cancer Institute, Erasmus University Medical Center, Rotterdam, The Netherlands; 9 Department of Neurosurgery, Tel Aviv Medical Center, Tel Aviv, Israel; 10 Department of Neurosurgery, University Clinic Hamburg-Eppendorf, Hamburg, Germany; 11 Department of Neurosurgery, Amsterdam University Medical Center, Amsterdam, The Netherlands; 12 Department of Neurosurgery, National Neurologic Institute IRCCS C. Besta, Milan, Italy; 13 Department of Biotechnology and Biosciences, University of Milano-Bicocca, Milan, Italy

**Keywords:** Glioblastoma, BMP4, Clinical trial, CED

## Abstract

**Background:**

This Phase 1 study evaluates the intra- and peritumoral administration by convection enhanced delivery (CED) of human recombinant Bone Morphogenetic Protein 4 (hrBMP4) – an inhibitory regulator of cancer stem cells (CSCs) – in recurrent glioblastoma.

**Methods:**

In a 3 + 3 dose escalation design, over four to six days, fifteen recurrent glioblastoma patients received, by CED, one of five doses of hrBMP4 ranging from 0·5 to 18 mg. Patients were followed by periodic physical, neurological, blood testing, magnetic resonance imaging (MRI) and quality of life evaluations. The primary objective of this first-in-human study was to determine the safety, dose-limiting toxicities (DLT) and maximum tolerated dose (MTD) of hrBMP4. Secondary objectives were to assess potential efficacy and systemic exposure to hrBMP4 upon intracerebral infusion.

**Results:**

Intra- and peritumoral infusion of hrBMP4 was safe and well-tolerated. We observed no serious adverse events related to this drug. Neither MTD nor DLT were reached. Three patients had increased hrBMP4 serum levels at the end of infusion, which normalized within 4 weeks, without sign of toxicity. One patient showed partial response and two patients a complete (local) tumor response, which was maintained until the most recent follow-up, 57 and 30 months post-hrBMP4. Tumor growth was inhibited in areas permeated by hrBMP4.

**Conclusion:**

Local delivery of hrBMP4 in and around recurring glioblastoma is safe and well-tolerated. Three patients responded to the treatment. A complete response and long-term survival occurred in two of them. This warrants further clinical studies on this novel treatment targeting glioblastoma CSCs.

**Trial registration:**

: ClinicaTrials.gov identifier: NCT02869243.

**Supplementary Information:**

The online version contains supplementary material available at 10.1186/s12943-023-01835-6.

## Background

Glioblastoma is the most aggressive brain cancer. Over four decades, progress toward more effective therapies in this field has been limited. This situation, along with the idiosyncratic biology of glioblastoma, imposes a radical change in therapeutic development for this cancer [1]. The cells crucially involved in glioblastoma pathophysiology retain key properties of immature neural precursors and stem cells and undergo regulation by extrinsic factors. A pivotal role in stem cell niches and neural cell development [2] is played by Bone Morphogenetic Proteins (BMPs), now viewed as pathophysiologic targets in oncology. BMPs’ actions in nervous tissues are pleiotropic, unraveling from early development to adulthood. Notably, BMPs drive adult neural progenitors toward astrocytic differentiation at the expense of stemness [3]. A similar phenomenon occurs in human glioblastoma stem-like cells (GSCs) and in breast cancer and melanoma, in which BMP type 4 (BMP4) reduces transformed stem cell pools [4]. This activity underlies BMP4’s ability to enforce pro-differentiation programs in glioblastomas, yielding potent anti-tumor effects, as observed in glioblastoma rodent models carrying patient-derived orthotopic implants of GSCs [5].

The findings above provide the rationale for a BMP4-driven, pro-differentiation clinical strategy for glioblastomas, tackling those key neurogenic mechanisms of malignant GSCs that are fundamental for their onset, maintenance, recurrence, and incurability. This innovative strategy, aimed at shrinking the stem cell pool that sustains glioblastoma’s growth, simultaneously increasing the efficacy of adjuvant therapies [6], underlies the first-in-human trial reported here (schematic overview in Supplementary Fig. 1). This trial evaluated the intra- and peritumoral administration of human recombinant (hr)BMP4 in recurrent glioblastoma patients.

## Methods

### Study design and participants

This was a prospective, international, multi-center, open-label, Phase 1 dose escalation trial on recurrent glioblastoma patients, evaluating safety and feasibility of hrBMP4 administered through convection enhanced delivery (CED). Secondary objectives were to assess systemic exposure to hrBMP4 upon intracerebral delivery and its efficacy. To achieve maximum tumor cell exposure and minimal systemic toxicity, hrBMP4 was administered intratumorally and intraparenchymally by CED. Since liquids spread poorly within glioblastoma, this technique consists of a slow continuous infusion with a positive pressure gradient, allowing hrBMP4 to better reach the tumor [7]. This study was approved by the Institutional Review Boards (IRBs) of the Erasmus University Medical Center (Rotterdam), the Amsterdam University Medical Center (Netherlands), the Tel Aviv Sourasky Medical Center (Israel), and the Neurological Institute “Carlo Besta” (Milan, Italy). Eligible patients, enrolled after written informed consent according to the Declaration of Helsinki, were ≥ 18 years of age, with a confirmed glioblastoma recurrence post-chemo-radiation therapy, and a Karnofsky Performance Score > 70. Tumors had to be unilateral, causing a limited mass effect with midline shift of ≤ 0·5 cm. Patients with corticosteroid dependency > 4 weeks, hematological disfunction (defined as White Blood Cell (WBC) count lower than 3.0 × 10^9^/L and Absolute Neutrophil Count (ANC) lower than 1.5 × 10^9^/L), liver or renal dysfunction were excluded. Pregnant women or of reproductive age and not using birth control were also excluded. Survival follow-ups were every two months, until death, or three years from the start of study treatment.

### CED catheter implantation

Preoperative magnetic resonance imaging (MRI) was used to plan catheter placement. Using iPlan Flow software (Brainlab AG Munich), drug infusion was simulated and catheter target positions optimized for maximal coverage of both tumor and surrounding parenchyma. Three modified silicone ventricular catheters (Medtronic) were used for CED and placed according to guidelines [8] under general anesthesia, using neuronavigation (Brainlab VarioGuide; see Fig. 3H). Some patients underwent resection of the tumor recurrence, followed by CED catheter placement around the resection cavity 1‒2 weeks later (or in one case 5 days before resection). Other patients underwent needle biopsy and placement of CED catheters in and around the recurring tumor in one surgical procedure. The location of the catheters and potential complications of their placement were assessed by CT scan before hrBMP4 infusion. Both 24 h of CED and at the end of infusion, T1-weighted non-intravenous-contrast MRI scans were performed to evaluate the distribution of the study drug.

### Drug preparation and dose escalation

hrBMP4 was purified from a working cell bank of CHO (Chinese Hamster Ovary) DGG cells (GEQA1058-2 cell line), producing hrBMP4 via a proprietary expression vector (pPGIX) by IBI Lorenzini (Aprilia; Italy). GMP certification for human use was obtained by AIFA (Authorization aM-188/2015). The study drug solution was prepared by dissolving hrBMP4 in sterile saline (NaCl 0·9%) up to a volume of 44–66 ml. Gadobutrol 1·0 mM was added to determine the study drug distribution in MRI scans. Increasing hrBMP4 doses were infused in five cohorts of three patients, from 0·5 to 18 mg from the first to the last cohort. The total volume was infused via CED catheters over 4 or 6 days (11 ml/day; 6 days for the 18 mg dose) with flow rates between 2·55 and 7·6 µL/minute, depending on dose level and number of catheters. If toxicity was observed in one out of three patients in a dose level, the other three patients would be treated with the same dose. In the absence of new toxicities, the next dose level would be assessed; if another toxicity occurred, the dose below would deemed to be the maximum tolerated dose (MTD).

### Safety assessments

During hospitalization, patients underwent daily physical and neurologic exams (including vital signs), hematology, blood chemistry, and urine tests, registration of adverse events (AEs) and concomitant medication use. This was repeated at 4, 8, 12, 24, 36, and 52 weeks post-discharge. AE severity was graded according to the NCI Common Terminology Criteria (CTC) for AEs, version 4.0, and the European Organization for the Research and Treatment of Cancer Quality of Life (QOL) Questionnaire (EORTC QLQ-30) [9] was completed by patients while hospitalized and 4, 8, 12, and 52 weeks post-treatment.

### Radiological assessment

At screening, during hospitalization and at 4, 12, 24, 36, and 52 weeks post-inclusion, MRI scans were performed, including T1-weighted pre- and post-contrast, T2-weighted, and T2-weighted FLAIR scans to assess tumor response by the MacDonald criteria [10]. Apparent diffusion coefficients (ADC) were calculated to improve radiological analyses and to flag underlying biological processes, such as cell proliferation.

### Statistics

We used Kaplan-Meier analysis to summarize the time-to-event variables, progression-free survival (PFS), and overall survival (OS). PFS was the time from treatment initiation to the first disease progression. Patients lost to follow-up were censored. No formal statistical calculation of sample size was made as the study followed a standard ‘3 + 3’ dose escalation design for 5 doses. A sample size of 3 evaluable patients per cohort was deemed a clinically reasonable sample. Approximately 18 evaluable patients were estimated.

## Results

### Patient demographics

Fifteen patients with histologically confirmed recurrent glioblastoma were recruited from 3 centers (Neurological Institute “Carlo Besta”, Erasmus Medical Center, and Amsterdam Medical Center) between July 2017 and October 2019. They were assigned to five cohorts of three patients, receiving increasing doses of hrBMP4 (0·5, 1·5, 4, 9, and 18 mg) via CED and included in the statistical analyses. Patient characteristics are shown in Supplementary Tables 1, Supplementary Tables 2 and Supplementary Fig. 2. Median age was 56 years (range: 33–70) and all patients had completed first-line standard chemoradiation (radiotherapy with concomitant temozolomide). Four patients had been treated with second-line chemotherapy upon first recurrence (lomustine, three patients; temozolomide, one patient). Seven patients underwent resection of the recurring tumor before hrBMP4, another patient received hrBMP4 followed by resection of the recurring tumor 5 days later.

### Pharmacokinetics, safety and tolerability

As shown in Supplementary Fig. 3 and in Supplementary Tables 3, in 12 patients, hrBMP4 serum levels did not change significantly after treatment. In patients 9, 10, and 15, hrBMP4 serum levels had a 0·5-, 1·9- and 0·5-fold increase compared to pre-treatment levels; they returned to pre-treatment values 4 weeks later. None of them experienced more AEs than the other patients. Both treatment protocol and the drug proved safe and well-tolerated. Neither dose-limiting toxicities (DLT) nor MTD were reached. A total of 97 AEs occurred (Supplementary Table 4) without any serious AEs (SAEs) or Suspected Unexpected Serious Adverse Reaction (SUSARS) related to hrBMP4 treatment. The AEs that occurred most frequently were headache (60%), vomiting (33%), hemiparesis (26·7%), and hyperglycemia (26·7%), the first two probably related to intracranial volume expansion due to CED as they mostly occurred < 2 weeks after catheter placement, and the latter to dexamethasone. Half of the four hemiparesis or worsening of pre-existing hemiparesis cases occurred within days from post-catheter placement and were possibly related to the surgical procedure. The other half were likely due to tumor progression. As expected, most AEs occurring later were related to clinical deterioration from glioblastoma progression. Supplementary Table 5 provides a detailed summary of AEs. Most AEs were either mild (grade 1, 50·5%) or moderate (grade 2, 26·8%) in severity. Of the 97 AEs reported, eight events in five patients were possibly related to hrBMP4, namely: lymphopenia (reported in two patients who had mild lymphopenia prior to hrBMP4 treatment), headache (n = 2), disorientation (n = 1), somnolence (n = 1), vomiting (n = 1, “probably related”), and wound pain (n = 1, “probably related”). All related events were grade 1 or 2, except lymphopenia which was grade 3. Unrelated SAEs were tumor progression (n = 12), euthanasia after established tumor progression (n = 2), wound infection (of resection wound, n = 1), and epileptic seizure (n = 1).

### Quality of life

Study participants had limited life expectancy and no therapeutic options. Mean global QOL was 63 (ranging 8 to 83, out of 100, during hospitalization) and fluctuated around this value during follow-up (Supplementary Table 6). QOL scores were relatively stable during treatment and seemed unrelated to hrBMP4 or its dose. Scores related to symptom scales like fatigue, nausea, and pain were higher during hrBMP4 infusion, decreasing to 0 (four out of four patients) 8 weeks post-discharge.

### Clinical and radiological follow-up

**Fig. 1:**
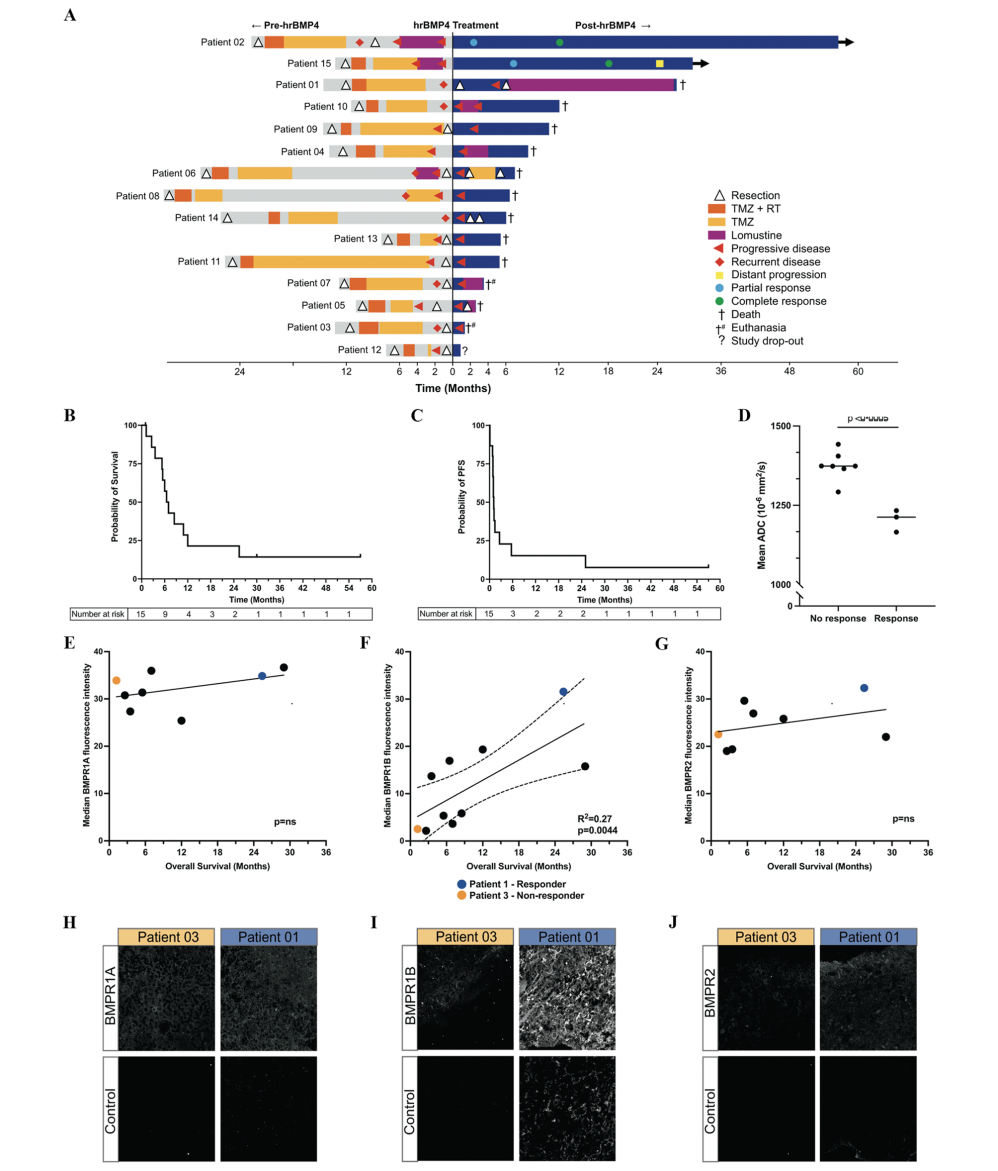
– Overview of patient survival and progression. **(A)** Survival after therapy with hrBMP4 depicted in a swimmer plot, TMZ = temozolomide, RT = radiotherapy. Treatment modalities and survival duration before treatment with hrBMP4 are also shown. **(B)** Kaplan-Meier estimates of OS and **(C)** PFS in months. **(D)** Mean ADC on preoperative MRI in patients with and without early tumor progression after hrBMP4 treatment. Median intensity of **E** BMPR1A, **F** BMPR1B, **G** BMPR2 in tumor tissue before treatment with hrBMP4 in relation to the OS of the respective patients. Representative images form patient 3 (orange) and patient 1 (blue) of staining with **H** BMPR1A, **I** BMPR1B and **J** BMPR2 and the respective negative-control samples only stained with secondary antibody

A detailed overview of all pre- (left, in gray) and post-hrBMP4 (right, in blue) treatments per each patient is shown by swimmer plot in Fig. 1A. The follow-up period happened between January 2018 and November 2020. The median PFS and OS were 1·2 and 7·0 months (Fig. 1B-C). Also, ADC values on pre-treatment MRI images, a marker associated with tumor cell density, were significantly lower in the three patients with the best PFS compared to the other patients (p = 0·0004) (Fig. 1D). When BMPR1A, BMPR1B and BMPR2 levels in tumor tissues before hrBMP4 treatment were analyzed, a significant association between BMPR1B expression and patient outcome emerged (R^2^ = 0.27, p = 0.0044) (Fig. 1E-J). Also, dataset analysis might suggest a possible association between longer patients’ survival and high mRNA expression of BMP4 concurrent with BMPR1A. Concurrent low BMP4 and BMPR1A levels seem to predict worse outcomes. Yet, a similar, homogeneous situation does not emerge for BMP4 and BMPR1B or BMPR2 for all gliomas (Supplementary Fig. 4).

**Fig. 2:**
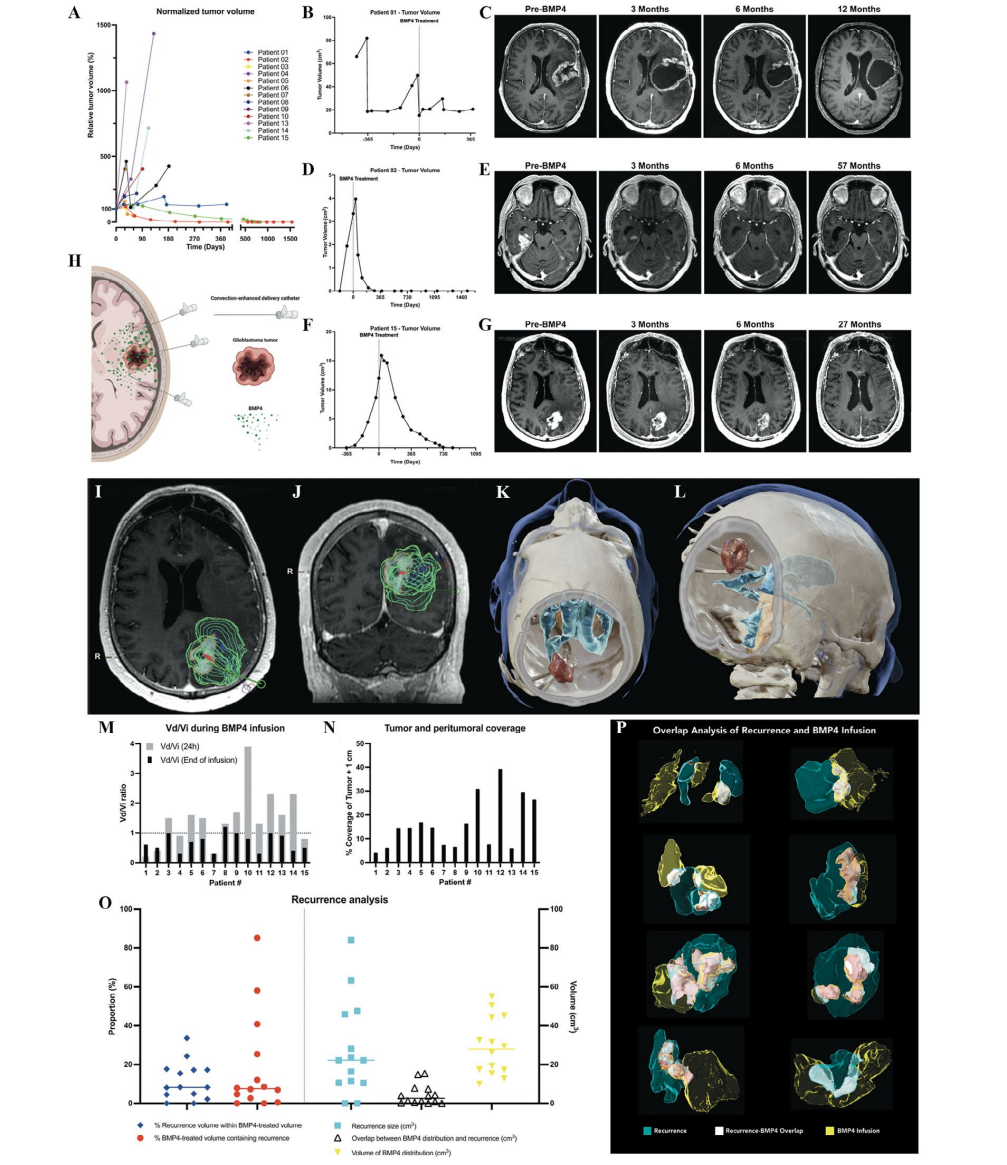
– Response, recurrence, and CED analysis. **(A)** Relative tumor volume normalized to the moment of study inclusion of all treated patients with available data. **(B, D, F)** Individual patient data showing tumor volume in the three patients showing response or stable disease (patient 1, 2, and 15), before and after hrBMP4 treatment. The dotted line indicates the moment of treatment with hrBMP4. **(C, E, G)** Representative images of the three patients with the best therapeutic response before study inclusion and at 3, 6- and 9-month follow-up. **(H)** Illustration of hrBMP4 CED concept. **(I, J)** Preoperative drug distribution planning, planning the simulated drug distribution over time (concentric green rings). **(K, L)** Postoperative 3D reconstructions showing tumor (red), CED catheters (white), and the ventricular system (blue). **(M)** Vd/Vi at 24 h of hrBMP4 infusion (gray) and at the end of infusion (black). **(N)** Coverage of tumor and peritumoral volume at the end of hrBMP4 infusion. **(O)** Analysis of recurrence location in relation to the volume of hrBMP4 infusion, showing the limited overlap (white) between hrBMP4 treated parenchymal volume (yellow) and recurrence volume (light blue). **(P)** Representative examples of overlap (white) between gadolinium/hrBMP4 distribution volume (yellow) and recurrence volume (blue), showing that the recurrent tumors largely grew outside of the hrBMP4 treated volume (colors match those in Fig. 2O).

As shown in Fig. 2A, some patients had early disease progression. Particularly, patient 1 experienced a partial response prior to progression at the 6-month MRI (Fig. 2B). Although the total non-enhancing lesion volume did not decrease substantially, there was a gradual decrease in contrast enhancement during the first 6 months (Fig. 2C). This patient underwent the due tumor resection 5 days after BMP4 infusion, and was treated with lomustine at progression, resulting in prolonged survival, totaling 25 months post-hrBMP4 treatment. Notably, two patients, one from the lowest (2) and one from the highest (15) hrBMP4 dose group, had a complete radiologic local tumor response without resection or second- or third-line treatment and were alive 57 and 30 months post-treatment. Before the study, patient 2 was treated for a right temporal glioblastoma, first with standard treatment (resection, radiotherapy, and temozolomide) and with resection followed by lomustine upon first recurrence. The second recurrence was treated with 0·5 mg of hrBMP4 followed by a steady, progressive decrease in tumor volume over 12 months, with no later recurrence (Fig. 2D-E). At the 57-month follow-up, this patient was clinically well, with improved motor function, stability, and gait compared to before hrBMP4 (data not shown). Patient 15 was initially treated for a left frontal glioblastoma with standard therapy, followed by recurrence with a distant lesion in the left parietal lobe that progressed despite lomustine, whereas the frontal lesion was stable and in remission. The parietal lesion was treated with hrBMP4 18 mg resulting in a slow regression of the tumor over 18 months, with a complete radiological response at that time (Fig. 2F-G). The patient’s first lesion in the frontal lobe progressed, while the treated parietal lesion remained in complete regression at the 30-month follow-up.

hrBMP4 treatment was given to 11 patients at first disease progression and to four at the second progression. Notably, the two complete responders (patients 2 and 15) belong to this latter group.

### Convection enhanced delivery (CED)

Pre-treatment CED flow simulation and catheter target and trajectory planning were performed in all patients (example in Fig. 2I-L). The ratio between distributed volume (Vd) and total infused volume (Vi) at the end of infusion ranged from 0·3 to 1·1, with a mean Vd/Vi of 0·7 (Fig. 2M-N). Convective spread resulting from CED was measured by MRI, assessing the volume of co-infused gadolinium based on 3D T1-weighted images performed prior, at day 1, and at the end of infusion, using a customized subtraction method. For each patient, the tumor volume was determined as the volume of contrast-enhancing tissue augmented by a 1 cm rim of tumor-infiltrated margin based on 3D T1-weighted MRI at baseline. Gadolinium distribution inside the tumor volume was considered as coverage and reported as a percentage of the tumor volume. The contrast enhancing tumor volume augmented by a 1 cm rim of tumor-infiltrated tissue ranged from 27·5 to 125·5 cm^3^ with a mean of 79·6 cm^3^. The percentage of tumor coverage by CED ranged from 4·2 to 39·3%, with a mean of 16%. (Fig. 2M-N). Quite notably, tumor recurrence after hrBMP4 occurred predominantly outside of the regions covered by CED and was rarely observed in the treated areas (Fig. 2O-P). Median recurrence volume was 22·2 cm^3^ (95% CI 10·5–47·6 cm^3^). Of this volume, a median of 2·6 cm^3^ (95% CI 0·2–7·9 cm^3^) was reached by gadolinium. Thus, recurrence was only observed in 8·3% (95% CI 2·1–17·7%) of the parenchyma reached by hrBMP4 (Fig. 2O).

## Discussion

This Phase 1 trial with hrBMP4 locally administered in 15 recurrent glioblastoma patients is the first clinical study on pro-differentiation therapy (PDT) in glioblastoma. Local administration of hrBMP4 by CED into the tumor and surrounding brain parenchyma was well-tolerated and safe, without drug-related, severe AEs. Three patients showed durable tumor response, including two patients with complete, sustained regression of the treated tumor and extended survival.

The first objective of this trial was to assess the safety and tolerability of intracerebral, local delivery of increasing doses of hrBMP4. This treatment did not cause any drug-related SAEs and no DLTs were observed. Most AEs were mild to moderate in severity and were related to the clinical deterioration typical of recurrent glioblastoma or to other factors, like concomitant medication. The reported headaches are common in trials with intracerebral local drug delivery and they resolved post-infusion. Most treatment-emergent AEs (TEAEs) surfaced in similar proportions in patients across all hrBMP4 dose levels, with no apparent dose-dependent trends in their number or severity. Accordingly, the three patients that had increased systemic levels of hrBMP4 after infusion did not have more or different AEs than patients retaining baseline systemic levels. Lymphopenia, one of grade 3 AEs observed in two patients, was an exacerbation of pre-existing conditions (grade 2) in both patients and no direct correlation between the latter and the circulating BMP4 levels (Supplementary Fig. 3) could be observed. Global QOL scores during hospitalization were similar to those of patients recently diagnosed with glioblastoma and patients receiving chemoradiation [11]. During hrBMP4 infusion, scores related to the symptom scales were relatively high compared to recurrent glioblastoma patients in other trials. For example, the mean score for constipation and nausea in our study were higher than those of patients in similar trials [12]. This is likely related to the surgical procedure, general anesthesia and hospitalization during CED, since scores normalized post-discharge, often reaching zero. Thus, hrBMP4 local delivery in the tumor and surrounding infiltrated brain by CED appears to be a safe and well-tolerated therapeutic procedure. Furthermore, considering that the duration of the infusion was ≤ 6 days and that no treatment discontinuations took place, the CED’s specific safety profile appears benign. Other trials using CED used shorter infusions. Thus, the apparent safety of long-term infusion may broaden the options for intraparenchymal drug delivery to the brain. We concluded that hrBMP4 infusion can be safely performed in recurrent glioblastoma patients.

The secondary objective of this trial was to assess the perspective efficacy of hrBMP4. While no standard treatment has yet been established for glioblastoma recurrences, lomustine is frequently used, leading to a median survival time of 5·6 months. The overall median survival time in our study was 7 months. The potentially beneficial role of hrBMP4 on survival was supported by the analysis of subgroups of responsive patients. Three out of 15 patients (20%) showed either partial response or complete local remission. Although over-interpretation of such results from early-phase trial should be avoided, “subgroup efficacy” is hypothesis-generating, in line with previously published in vitro studies that also proposed subgroup efficacy after treatment with hrBMP4 [13]. Both complete responders had IDH1 wildtype tumors and did not receive any additional treatment throughout follow-up, suggesting that their response was due to the interaction between the tumor cells and hrBMP4. This is further supported by the high tumoral mRNA expression of BMPR1B, a high affinity BMP4 co-receptor that generally correlates with increased patient survival (Fig. 1F). Notably, recurrence post-hrBMP4 treatment occurred predominantly in areas not covered by the drug infusion (Fig. 2N, O). This coincides with the anticipated actions of BMP4 on glioblastoma cells, particularly with the expected shrinkage of the glioblastoma GSC pool upon exposure to this pro-differentiation protein. Furthermore, imaging assessments showed that lower ADC was significantly associated with a good PFS/OS. However, previous research has shown that a low ADC is associated with unfavorable prognosis and a high cell density and number of proliferating cells [14]. As one of the main actions of BMP4 is to induce reduced mitotic activity by differentiation, its effects might be more profound in tumors with high proliferation rates [6]. In future studies, it will be important to try to determine the perspective effects of BMP4 infusion on the GSCs differentiation status, also in consideration of the fact that, at least in some instances, BMPs might underpin proliferative effects on GSCs [15] or lead to GSCs quiescence [16], which might have an impact on long-term recurrences following treatment.

Altogether, these findings support the concept that the infusion of hrBMP4 via CED can be safely conducted and may elicit therapeutic effects in glioblastoma patients.

One of the limitations of this study was one of the inherent issues with CED, i.e. the difficulty in anticipating the infusate distribution. Especially for recurrent tumors, which have rubbery gliotic boundaries, areas of necrosis, and dispersed islands of rapidly proliferating tissue, optimal target delineation for catheters is challenging. Hence, we analyzed the distribution of the infused hrBMP4 in relation to the patient’s lesions and pattern of disease progression. This revealed that the hrBMP4-treated areas rarely overlapped with those wherein the disease recurred (Fig. 2N, O). Also, the two patients that experienced complete and durable local response and extended survival were among those in which we achieved a relatively high coverage of the target lesion (Fig. 2M, N). In future trials, the efficacy of this treatment may be increased by using new, dedicated CED catheters and strategies that increase lesion coverage up to 90%, permitting both prolonged infusion and re-treatment. This may also include pre-resection CED allowing robust distribution within the peritumoral non-resectable surroundings, minimizing the risk of infusion leakage.

Another limitation was that, as typical for a hypothesis-generating Phase I trial, the enrollment criteria did not discriminate between molecular glioblastoma subtypes. Since strong BMPR1B signals and low ADC values may be associated with better survival after hrBMP4, these parameters should be investigated to guide patient stratification.

In conclusion, hrBMP4 through CED is safe and well-tolerated in patients with recurrent glioblastoma. While efficacy was not the primary aim of this study, there was a response signal with significant tumor reduction or complete response in three out of 15 patients. Complete remission occurred in the absence of any other continued treatment and tumor appears not to grow in tissue reached by hrBMP4. This supports the rationale for pro-differentiation therapy in glioblastoma and encourages the conduct of Phase 2 trials powered to define the efficacy of hrBMP4 delivered through CED.

## Conclusion

Prolonged intraparenchymal infusion of the pro-differentiation cytokine hrBMP4, targeting GSCs, was safe and without drug-related severe adverse events in glioblastoma recurrences. Three patients exhibited durable tumor response, two of which showed sustained, complete regression of the treated tumor and extended survival. Tumor growth was greatly reduced in areas permeated by hrBMP4. This supports the implementation of Phase 2 trials powered to validate the efficacy of this pro-differentiation treatment in glioblastoma.

### Electronic supplementary material

Below is the link to the electronic supplementary material.

Supplementary Material 1

Supplementary Material 2

Supplementary Material 3

## Data Availability

All individual participant data that underlie the results reported in this article, after de-identification will be available immediately after publication (no end date) upon reasonable request to the corresponding author.

## Declarations

### Declarations

Eligible patients were enrolled and signed informed consents were collected according to the ethical guidelines of the 2013 Declaration of Helsinki. This study was approved by the Institutional Review Boards (IRBs) of the Erasmus University Medical Center (Rotterdam), the Amsterdam University Medical Center (Netherlands), the Tel Aviv Sourasky Medical Center (Israel), and the Neurological Institute “Carlo Besta” (Milan, Italy). All authors approved the manuscript and gave their consent for submission and publication.

## List of abbreviations

CED: convection enhanced delivery
hrBMP4: human recombinant Bone Morphogenetic Protein 4
CSCs: cancer stem cells
MRI: magnetic resonance imaging
DLT: dose-limiting toxicities
MTD: maximum tolerated dose
GSCs: glioblastoma stem cell-like cells
AEs: adverse events
SAEs: serious AEs
ADC: Apparent diffusion coefficients
QOL: Treatment of Cancer Quality of Life
PFS: progression-free survival
OS: overall survival
CNV: Copy Number Variation
PDT: pro-differentiation therapy
