# Supplementary material for: Local delivery of hrBMP4 as an anticancer therapy in patients with recurrent glioblastoma: a first-in-human phase 1 dose escalation trial
Source: Mol Cancer. 2023 Aug 10;22:129. doi: 10.1186/s12943-023-01835-6 (PMC10413694; doi:10.1186/s12943-023-01835-6)
Supplement: Supplementary file 1 — Supplementary Material 1 [file 12943_2023_1835_MOESM1_ESM.docx]

**Pro-differentiation anticancer therapy using local delivery of hrBMP4 in patients with recurrent glioblastoma: a first-in-human Phase 1 dose escalation trial**

Eelke M. Bos*^1^, Elena Binda*^2^, Iris S.C. Verploegh*^1,3^, Eva Wembacher^4^, Daphna Hoefnagel^1^, Rutger K. Balvers^1^, Anne L. Korporaal^2^, Andrea Conidi^2^, Esther A. H. Warnert^5^, Nadia Trivieri^2^, Alberto Visioli^11,12^, Paola Zaccarini^11,12^, Laura Caiola^11,12^, Rogier van Wijck^6^, Peter van der Spek^6^, Danny Huylebroeck^2^, Sieger Leenstra^1^, Martine L.M. Lamfers^1^, Zvi Ram^7^, Manfred Westphal^8^, David Noske^9^, Federico Legnani^10^, Francesco DiMeco^10^, Angelo L. Vescovi^#§2,13^, Clemens M.F. Dirven^§1^

^1^Department of Neurosurgery, Erasmus MC Cancer Institute, University Medical Center Rotterdam, The Netherlands

^2^Unit of Cancer Stem Cells, ISBReMIT, IRCCS Casa Sollievo della Sofferenza, San Giovanni Rotondo (FG), Italy

^3^Department of Cell Biology, Erasmus MC, University Medical Center Rotterdam, The Netherlands

^4^Brainlab A.G., Munich, Germany

^5^Department of Radiology, Erasmus MC Cancer Institute, University Medical Center Rotterdam, The Netherlands

^6^Department of Clinical Bioinformatics, Erasmus MC, University Medical Center Rotterdam, The Netherlands

^7^Department of Neurosurgery, Tel Aviv Medical Center, Israel

^8^Department of Neurosurgery, University Clinic Hamburg-Eppendorf, Hamburg, Germany

^9^Department of Neurosurgery, Amsterdam University Medical Center, The Netherlands

^10^Department of Neurosurgery, Istituto Neurologico C. Besta, Milan, Italy

^11^ StemGen SpA, Milan, Italy

^12^ HyperStem SA, Lugano, Switzerland

^13^ Department of Biotechnology and Biosciences University of Milano-Bicocca, Milan, Italy

^#^**Corresponding author:**

Angelo Luigi Vescovi

IRCCS Casa Sollievo della Sofferenza, Scientific Directorate

Viale dei Cappuccini 1, 71013, San Giovanni Rotondo (FG), Italy

E-mail: [angelo.vescovi@unimib.it](mailto:angelo.vescovi@unimib.it)

**SUPPLEMENTARY MATERIALS**


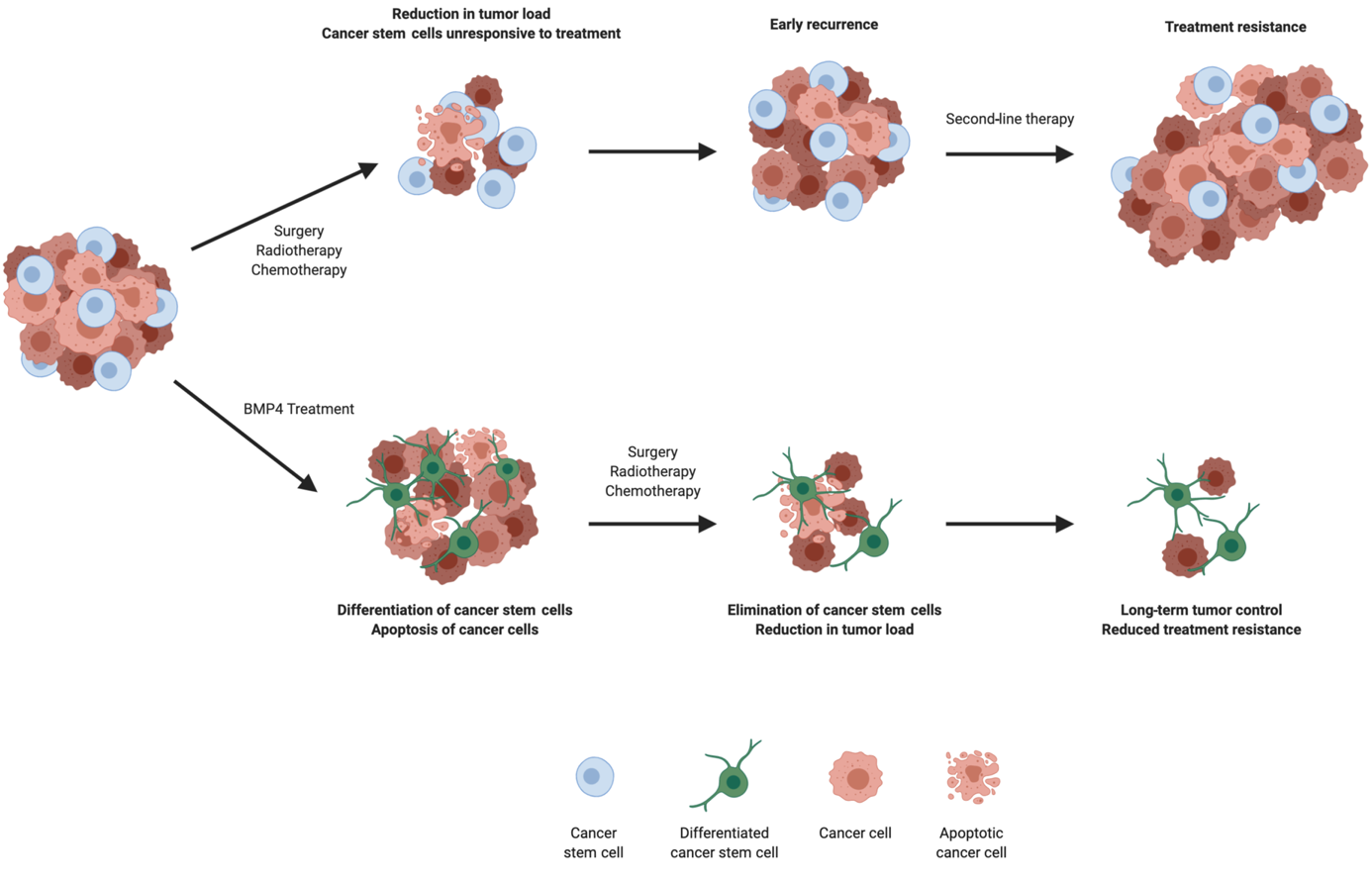


**Supplementary Figure S1**: Hypothetical schema of BMP4’s mechanism of action as a PDT.


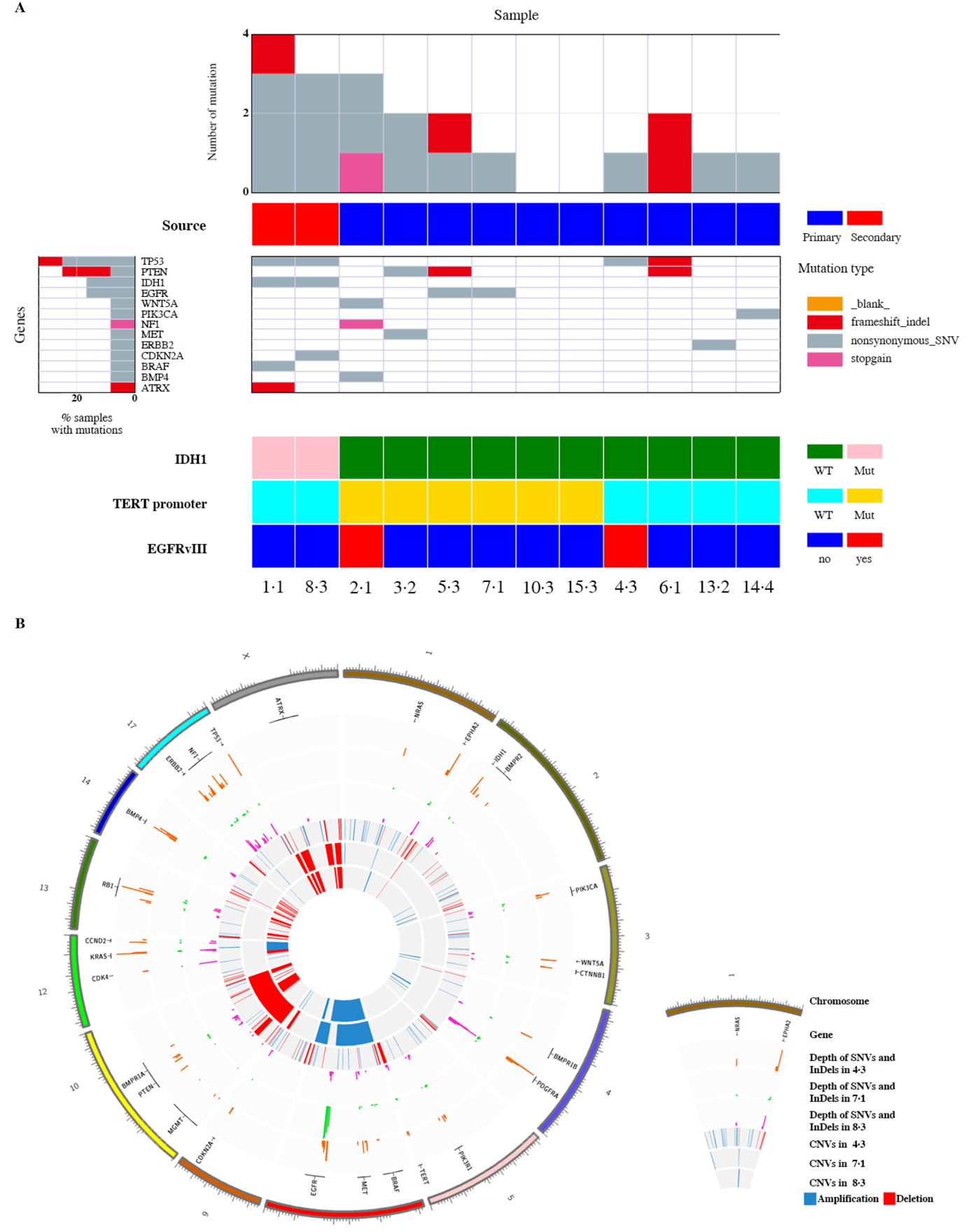


**Supplementary Figure S2**: Genetic analysis of tumors from patients included in this study. (A) Overview of mutations found in the treated patients. (B) Circos plot for genetic alterations detected in our samples. The outer track provides somatic single nucleotide variants (SNVs), small insertions, and deletions (indels); and the inner track CNVs. -log10 (q-value) of the significant amplified or deleted region in blue or red, respectively.

**
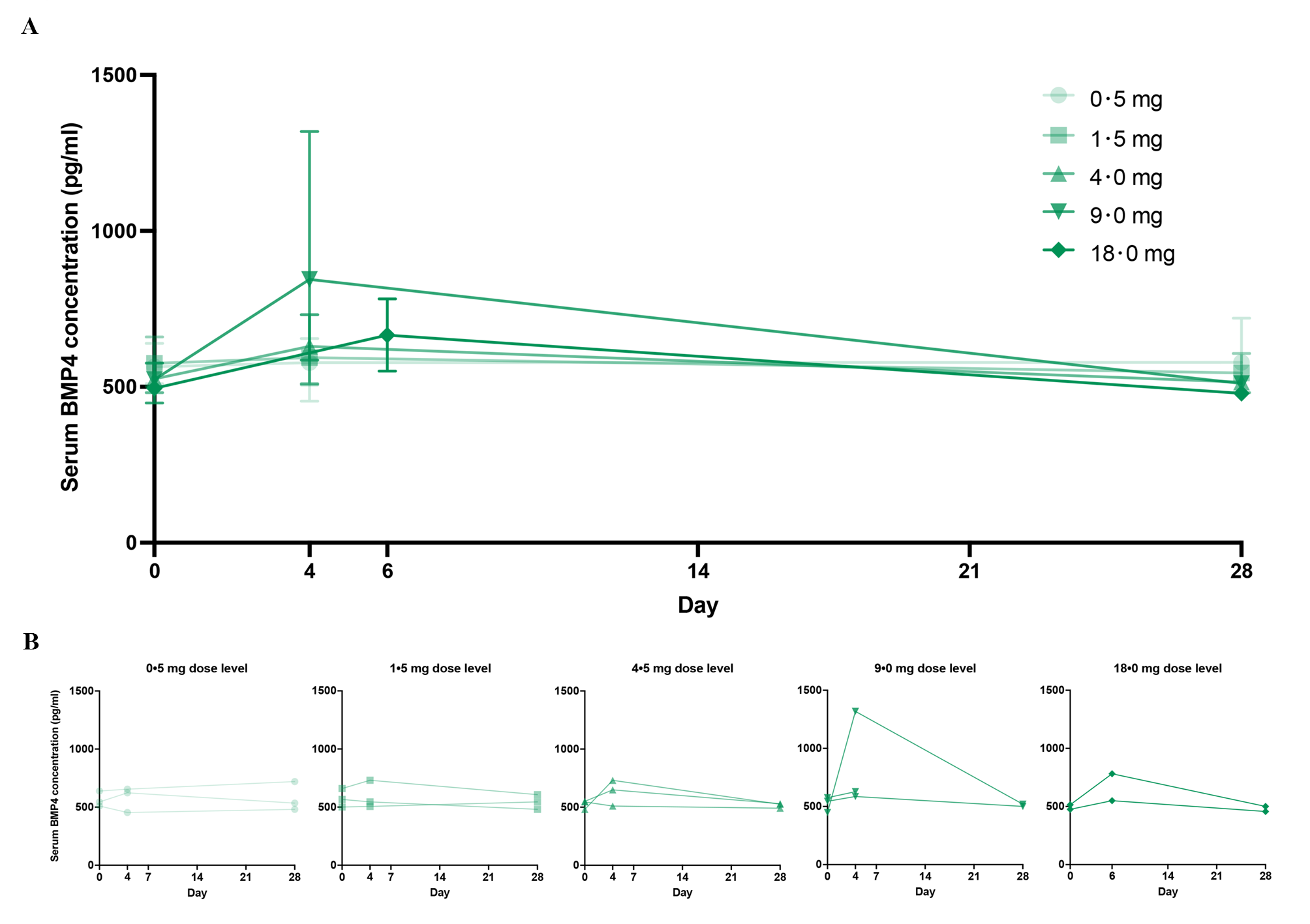
**

**Supplementary Figure S3.** (A) Serum levels of hrBMP4 in pg/ml at baseline (day 0), at the end of infusion with hrBMP4 (day 4 or 6, depending on dose level), and 4 weeks after infusion. Data is split by dose level. (B) Individual patient data within each dose-level cohort, showing serum levels of hrBMP4 before infusion, at the end of infusion, and 4 weeks after treatment in the different dose levels.

.


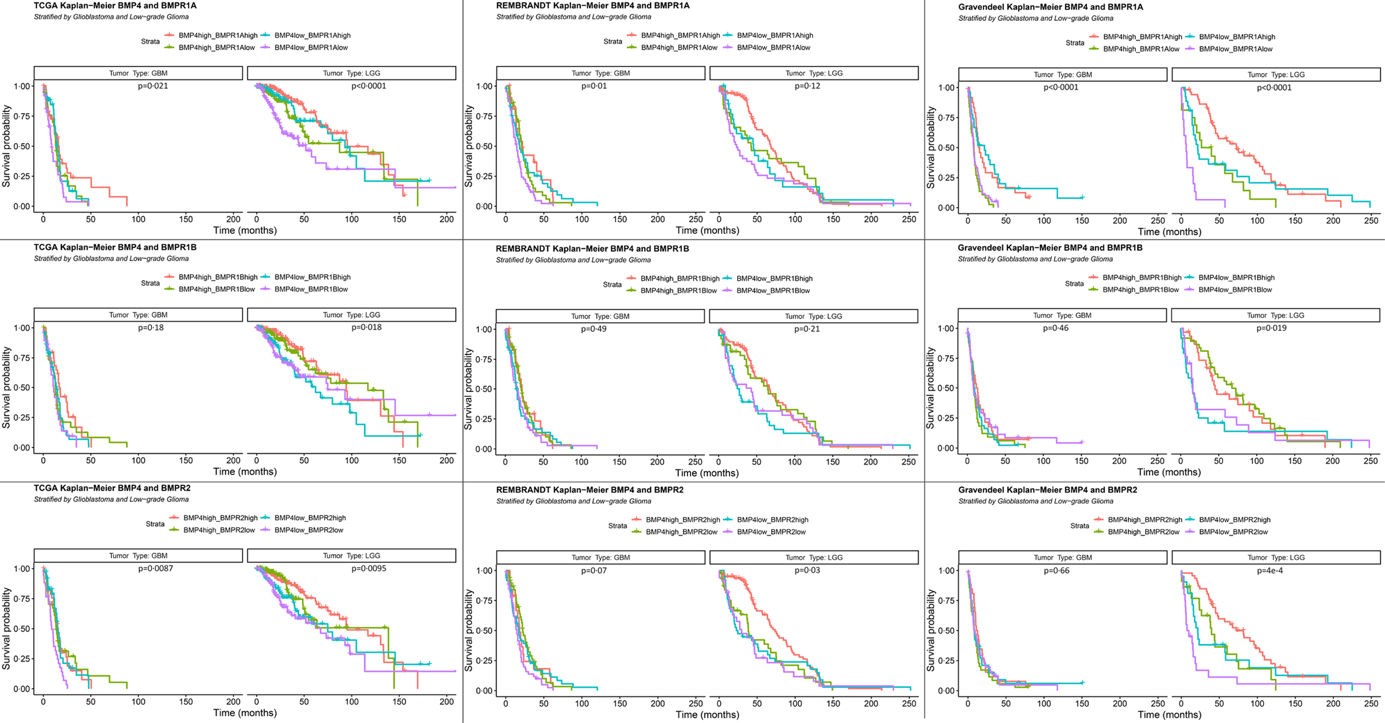


**Supplementary Figure S4.** Kaplan-Meier survival plots for low- and glioblastoma multiforme (LGG and GBM) in three independent public glioma datasets (TCGA, REMBRANDT and Gravendeel) showing the increased expression of BMP4 and its BMPR1A receptor is associated with a better prognosis in low- and high-grade glioma patients.

**SUPPLEMENTARY TABLES**

| **Patient** | **Dose (mg)** | **Age** | **Gender** | | **KPS** | | **Survival before trial start (months)** | **IDH1 mutant** | **RT+TMZ**  **(# TMZ cycles)*** | **Lomustine*** | **Re-resection*** | **Re-resection before hrBMP4** | | **Tumor location** | |
| --- | --- | --- | --- | --- | --- | --- | --- | --- | --- | --- | --- | --- | --- | --- | --- |
| 1 | 0∙5 | 63 | M | | 80 | | 15 | Y | Yes (6) |  |  |  | | Left Temporal | |
| 2 | 0∙5 | 56 | F | | 80 | | 22 | N | Yes (6) | x | x |  | | Right Temporal/Occipital | |
| 3 | 0∙5 | 51 | F | | 80 | | 13 | N | Yes (5) |  |  | x | | Right Frontal/Temporal/Parietal | |
| 4 | 1∙5 | 44 | F | | 100 | | 14 | N | Yes (6) |  |  |  | | Right Frontal | |
| 5 | 1∙5 | 56 | M | | 80 | | 11 | N | Yes (6) |  | x |  | | Right Frontal | |
| 6 | 1∙5 | 33 | F | | 90 | | 28 | N | Yes (6) | x |  | x | | Right Frontal | |
| 7 | 4 | 53 | M | | 90 | | 13 | N | Yes (5) |  |  | x | | Temporal/Parietal/Occipital | |
| 8 | 4 | 38 | M | | 90 | | 33 | Y | Yes (2) |  |  |  | | Left Frontal/Temporal/Parietal | |
| 9 | 4 | 40 | M | | 100 | | 14 |  | Yes (11) |  |  | x | |  | |
| 10 | 9 | 56 | M | | 90 | | 11 | N | Yes (5) |  |  |  | | Right Temporal | |
| 11 | 9 | 56 | M | | 90 | | 26 |  | Yes (21) |  |  | x | |  | |
| 12 | 9 | 64 | M | | 80 | | 8 |  | Yes (0) |  |  | x | |  | |
| 13 | 18 | 36 | M | | 90 | | 8 | N | Yes (2) |  |  | x | | Left Frontal/Parietal | |
| 14 | 18 | 70 | F | | 80 | | 26 | N | Yes (6) |  |  |  | | Left Temporal/Occipital | |
| 15 | 18 | 63 | M | | 90 | | 14 | N | Yes (5) | x |  |  | | Left Parietal | |
| *Before inclusion into this trial | | | |  | |  | | |  |  |  | |  | |  |

**Supplementary Table S1: Patient demographic characteristics**

**Supplementary Table S2: Tumor genetic characteristics**

| **Patient** | **BMP4 Dose**  **(mg)** | **GBM Subgroup** | **IDH1 mutant** | **TERT promoter mutation** | **EGFRvIII mutation** | **1p19q codeletion** | **CHR7**  **amplification** | **CHR10**  **deletion** | **Mutated Genes in NGS Panel** |
| --- | --- | --- | --- | --- | --- | --- | --- | --- | --- |
| 1 | 0∙5 | Secondary IDH1^MUT^-TERTp^WT^ | Y | WT | NO | NO | NO | NO | BRAF, TP53, ATRX |
| 2 | 0∙5 | Primary IDH1^WT^-TERTp^MUT^ | N | C228T | YES | NO | YES | YES | WNT5A, BMP4, TP53 |
| 3 | 0∙5 | Primary IDH1^WT^-TERTp^MUT^ | N | C228T | NO | NO | YES | YES | MET, PTEN |
| 4 | 1∙5 | Primary IDH1^WT^-TERTp^WT^ | N | WT | YES | NO | YES | YES | TP53 |
| 5 | 1∙5 | Primary IDH1^WT^-TERTp^MUT^ | N | C288T | NO | NO | YES | YES | EGFR, PTEN |
| 6 | 1∙5 | Primary IDH1^WT^-TERTp^WT^ | N | WT | NO | NO | YES | NO | PTEN, TP53 |
| 7 | 4 | Primary IDH1^WT^-TERTp^MUT^ | N | C288T | NO | NO | YES | YES | EGFR |
| 8 | 4 | Secondary IDH1^MUT^-TERTp^WT^ | Y | WT | NO | NO | YES | YES | CDKN2A, TP53 |
| 9 | 4 |  |  |  |  |  |  |  |  |
| 10 | 9 | Primary IDH1^WT^-TERTp^MUT^ | N | C250T | NO | NO | YES | YES |  |
| 11 | 9 |  |  |  |  |  |  |  |  |
| 12 | 9 |  |  |  |  |  |  |  |  |
| 13 | 18 | Primary IDH1^WT^-TERTp^WT^ | N | WT | NO | NO | NO | NO | ERBB2 |
| 14 | 18 | Primary IDH1^WT^-TERTp^WT^ | N | WT | NO | NO | YES | YES | PIK3CA |
| 15 | 18 | Primary IDH1^WT^-TERTp^MUT^ | N | C288T | NO | NO | YES | YES |  |

**Supplementary Table S3: Overview of reported adverse events**

|  | | | |  |  |  |
| --- | --- | --- | --- | --- | --- | --- |
|  | **Number of events** | | | | | |
| Event | 0∙5 mg (n=3) | 1∙5 mg (n=3) | 4∙0 mg (n=3) | 9∙0 mg (n=3) | 18 mg (n=3) | Total (n=15) |
| AEs | 19 | 13 | 20 | 17 | 28 | 97 |
| Related | 1 | 0 | 0 | 1 | 6 | 8 |
| SAEs | 3 | 3 | 4 | 3 | 4 | 17 |
| Related | 0 | 0 | 0 | 0 | 0 | 0 |
| DLT | 0 | 0 | 0 | 0 | 0 | 0 |

**Supplementary Table S4: Summary of Treatment Emergent Adverse Events (TEAE) by System Organ Class (in ≥ 25% of patients) and Preferred Term**

| **Overall** | **hrBMP4 0∙5 mg**  **(N=3)** | **hrBMP4 1∙5 mg**  **(N=3)** | **hrBMP4 4 mg**  **(N=3)** | **hrBMP4 9 mg**  **(N=3)** | **hrBMP4 18 mg**  **(N=3)** | **Overall**  **(N=15)** |
| --- | --- | --- | --- | --- | --- | --- |
|  | E n | E n | E n | E n | E n | E n (%) |
| **Any TEAE** | 19 3 | 13 3 | 20 3 | 17 3 | 28 3 | 97 15 (100∙0%) |
| **Blood and lymphatic system disorders** | 1 1 | - | - | 3 2 | 1 1 | 5 4 (26∙7%) |
| Anaemia | 1 1 | - | - | 1 1 | 1 1 | 3 3 (20∙0%) |
| Leukopenia | - | - | - | 1 1 | - | 1 1 (6∙7%) |
| Lymphopenia | - | - | - | 1 1 | - | 1 1 (6∙7%) |
| **Gastrointestinal disorders** | 2 1 | - | 1 1 | 1 1 | 4 3 | 8 6 (40∙0%) |
| Gastritis | - | - | - | 1 1 | - | 1 1 (6∙7%) |
| Nausea | 1 1 | - | - | - | 1 1 | 2 2 (13∙3%) |
| Vomiting | 1 1 | - | 1 1 | - | 3 3 | 5 5 (33∙3%) |
| **General disorders and administration site conditions** | 1 1 | - | 3 2 | - | 2 1 | 6 4 (26∙7%) |
| Catheter site haemorrhage | - | - | 1 1 | - | - | 1 1 (6∙7%) |
| Euthanasia | 1 1 | - | 1 1 | - | - | 2 2 (13∙3%) |
| Pain | - | - | - | - | 1 1 | 1 1 (6∙7%) |
| Pyrexia | - | - | 1 1 | - | 1 1 | 2 2 (13∙3%) |
| **Injury, poisoning and procedural complications** | 1 1 | - | 1 1 | - | 2 2 | 4 4 (26∙7%) |
| Wound | 1 1 | - | - | - | - | 1 1 (6∙7%) |
| Wound complication | - | - | 1 1 | - | 1 1 | 2 2 (13∙3%) |
| Wound infection | - | - | - | - | 1 1 | 1 1 (6∙7%) |
| **Investigations** | 5 2 | 2 1 | 6 2 | 2 1 | 5 2 | 20 8 (53∙3%) |
| Alanine aminotransferase increased | 1 1 | - | - | - | 1 1 | 2 2 (13∙3%) |
| Aspartate aminotransferase increased | 1 1 | - | 1 1 | - | 1 1 | 3 3 (20∙0%) |
| Blood bicarbonate increased | - | - | 1 1 | - | 1 1 | 2 2 (13∙3%) |
| Blood bilirubin increased | - | - | 1 1 | - | - | 1 1 (6∙7%) |
| Blood creatine decreased | - | - | - | - | 1 1 | 1 1 (6∙7%) |
| Blood creatinine increased | 1 1 | - | - | - | - | 1 1 (6∙7%) |
| Blood lactate dehydrogenase increased | - | 1 1 | 1 1 | 1 1 | - | 3 3 (20∙0%) |
| Gamma-glutamyltransferase increased | 1 1 | - | 1 1 | - | - | 2 2 (13∙3%) |
| Lymphocyte count decreased | 1 1 | - | 1 1 | 1 1 | 1 1 | 4 4 (26∙7%) |
| Red blood cell count decreased | - | 1 1 | - | - | - | 1 1 (6∙7%) |
| **Metabolism and nutrition disorders** | - | 2 1 | 2 2 | 1 1 | 4 2 | 9 6 (40∙0%) |
| Hyperglycaemia | - | 1 1 | 2 2 | - | 1 1 | 4 4 (26∙7%) |
| Hypoalbuminaemia | - | - | - | - | 1 1 | 1 1 (6∙7%) |
| Hypokalaemia | - | - | - | 1 1 | - | 1 1 (6∙7%) |
| Hypophosphataemia | - | 1 1 | - | - | 2 2 | 3 3 (20∙0%) |
| **Neoplasms benign, malignant and unspecified (incl cysts and polyps)** | 2 2 | 3 3 | 3 3 | 2 2 | 3 2 | 13 12 (80∙0%) |
| Neoplasm progression | 2 2 | 3 3 | 3 3 | 2 2 | 3 2 | 13 12 (80∙0%) |
| **Nervous system disorders** | 7 2 | 5 2 | 4 2 | 4 2 | 3 2 | 23 10 (66∙7%) |
| Aphasia | 1 1 | - | - | - | - | 1 1 (6∙7%) |
| Extensor plantar response | 1 1 | - | - | - | - | 1 1 (6∙7%) |
| Headache | 1 1 | 2 2 | 2 2 | 4 2 | 2 2 | 11 9 (60∙0%) |
| Hemiparesis | 2 2 | 1 1 | 2 1 | - | - | 5 4 (26∙7%) |
| Muscle spasticity | 1 1 | - | - | - | - | 1 1 (6∙7%) |
| Nervous system disorders | - | 1 1 | - | - | - | 1 1 (6∙7%) |
| Seizure | - | - | - | - | 1 1 | 1 1 (6∙7%) |
| Sensory loss | 1 1 | - | - | - | - | 1 1 (6∙7%) |
| Tremor | - | 1 1 | - | - | - | 1 1 (6∙7%) |

**Supplementary Table S5: Overview of Quality-of-Life reporting**

|  | | | | | | | | | | | | |
| --- | --- | --- | --- | --- | --- | --- | --- | --- | --- | --- | --- | --- |
|  | 0∙5 mg | | 1∙5 mg | | 4∙0 mg | | 9∙0 mg | | 18∙0 mg | | Total | |
| EORTC QLQ-C30 | Mean | n | Mean | n | Mean | n | Mean | n | Mean | n | Mean | n |
| **Functioning scales** |  |  |  |  |  |  |  |  |  |  |  |  |
| Physical |  |  |  |  |  |  |  |  |  |  |  |  |
| Hospitalization | 71 | 3 | 62 | 3 | 87 | 3 | 84 | 3 | 89 | 3 | 79 | 15 |
| Week 4 | 29 | 3 | 60 | 2 | 89 | 3 | 70 | 2 | 84 | 3 | 67 | 13 |
| Week 8 | 57 | 2 |  |  | 80 | 1 |  |  | 100 | 1 | 73 | 4 |
| Week 12 | 43 | 2 |  |  |  |  |  |  | 100 | 1 | 62 | 3 |
| Week 52 | 93 | 1 |  |  | 53 | 1 |  |  | 100 | 1 | 82 | 3 |
| Role |  |  |  |  |  |  |  |  |  |  |  |  |
| Hospitalization | 39 | 3 | 22 | 3 | 56 | 3 | 83 | 3 | 61 | 3 | 52 | 15 |
| Week 4 | 22 | 3 | 50 | 2 | 56 | 3 | 58 | 2 | 78 | 3 | 53 | 13 |
| Week 8 | 33 | 2 |  |  | 33 | 1 |  |  | 100 | 1 | 50 | 4 |
| Week 12 | 25 | 2 |  |  |  |  |  |  | 100 | 1 | 50 | 3 |
| Week 52 | 33 | 1 |  |  | 33 | 1 |  |  | 67 | 1 | 44 | 3 |
| Emotional |  |  |  |  |  |  |  |  |  |  |  |  |
| Hospitalization | 72 | 3 | 83 | 2 | 78 | 3 | 83 | 3 | 69 | 3 | 77 | 14 |
| Week 4 | 67 | 3 | 38 | 2 | 61 | 3 | 71 | 2 | 81 | 3 | 65 | 13 |
| Week 8 | 96 | 2 |  |  | 75 | 1 |  |  | 100 | 1 | 92 | 4 |
| Week 12 | 63 | 2 |  |  |  |  |  |  | 100 | 1 | 75 | 3 |
| Week 52 | 50 | 1 |  |  | 67 | 1 |  |  | 100 | 1 | 72 | 3 |
| Cognitive |  |  |  |  |  |  |  |  |  |  |  |  |
| Hospitalization | 61 | 3 | 44 | 3 | 83 | 3 | 72 | 3 | 67 | 3 | 66 | 15 |
| Week 4 | 50 | 3 | 33 | 2 | 61 | 3 | 67 | 2 | 67 | 3 | 56 | 13 |
| Week 8 | 50 | 2 |  |  | 100 | 1 |  |  | 83 | 1 | 71 | 4 |
| Week 12 | 33 | 2 |  |  |  |  |  |  | 83 | 1 | 50 | 3 |
| Week 52 | 67 | 1 |  |  | 100 | 1 |  |  | 83 | 1 | 83 | 3 |
| Social |  |  |  |  |  |  |  |  |  |  |  |  |
| Hospitalization | 56 | 3 | 50 | 3 | 94 | 3 | 78 | 3 | 89 | 3 | 73 | 15 |
| Week 4 | 56 | 3 | 33 | 2 | 78 | 3 | 58 | 2 | 94 | 3 | 67 | 13 |
| Week 8 | 33 | 2 |  |  | 83 | 1 |  |  | 100 | 1 | 63 | 4 |
| Week 12 | 33 | 2 |  |  |  |  |  |  | 100 | 1 | 56 | 3 |
| Week 52 | 50 | 1 |  |  | 83 | 1 |  |  | 83 | 1 | 72 | 3 |
| Global |  |  |  |  |  |  |  |  |  |  |  |  |
| Hospitalization | 50 | 3 | 69 | 3 | 75 | 3 | 72 | 3 | 50 | 3 | 63 | 15 |
| Week 4 | 36 | 3 | 58 | 2 | 61 | 3 | 58 | 2 | 67 | 3 | 56 | 13 |
| Week 8 | 54 | 2 |  |  | 33 | 1 |  |  | 62 | 1 | 58 | 4 |
| Week 12 | 50 | 2 |  |  |  |  |  |  | 83 | 1 | 61 | 3 |
| Week 52 | 83 | 1 |  |  | 33 | 1 |  |  | 83 | 1 | 67 | 3 |
| **Symptom scales** |  |  |  |  |  |  |  |  |  |  |  |  |
| Fatigue |  |  |  |  |  |  |  |  |  |  |  |  |
| Hospitalization | 37 | 3 | 61 | 2 | 41 | 3 | 26 | 3 | 52 | 3 | 42 | 14 |
| Week 4 | 63 | 3 | 72 | 2 | 37 | 3 | 50 | 2 | 30 | 3 | 49 | 13 |
| Week 8 | 56 | 2 |  |  | 22 | 1 |  |  | 11 | 1 | 36 | 4 |
| Week 12 | 50 | 2 |  |  |  |  |  |  | 0 | 1 | 33 | 3 |
| Week 52 | 22 | 1 |  |  | 56 | 1 |  |  | 0 | 1 | 26 | 3 |
| Nausea/vomiting |  |  |  |  |  |  |  |  |  |  |  |  |
| Hospitalization | 6 | 3 | 11 | 3 | 0 | 3 | 0 | 3 | 56 | 3 | 14 | 15 |
| Week 4 | 6 | 3 | 8 | 2 | 11 | 3 | 0 | 2 | 0 | 3 | 5 | 13 |
| Week 8 | 0 | 2 |  |  | 0 | 1 |  |  | 0 | 1 | 0 | 4 |
| Week 12 | 0 | 2 |  |  |  |  |  |  | 0 | 1 | 0 | 3 |
| Week 52 | 0 | 1 |  |  | 0 | 1 |  |  | 0 | 1 | 0 | 3 |
| Pain |  |  |  |  |  |  |  |  |  |  |  |  |
| Hospitalization | 22 | 3 | 39 | 3 | 28 | 3 | 6 | 3 | 22 | 3 | 23 | 15 |
| Week 4 | 33 | 3 | 25 | 2 | 28 | 3 | 8 | 2 | 11 | 3 | 22 | 13 |
| Week 8 | 0 | 2 |  |  | 0 | 1 |  |  | 0 | 1 | 0 | 4 |
| Week 12 | 0 | 2 |  |  |  |  |  |  | 0 | 1 | 0 | 3 |
| Week 52 | 0 | 1 |  |  | 0 | 1 |  |  | 0 | 1 | 0 | 3 |
| Dyspnea |  |  |  |  |  |  |  |  |  |  |  |  |
| Hospitalization | 22 | 3 | 44 | 3 | 0 | 3 | 0 | 3 | 11 | 3 | 16 | 15 |
| Week 4 | 22 | 3 | 0 | 2 | 0 | 3 | 33 | 2 | 0 | 3 | 10 | 13 |
| Week 8 | 0 | 2 |  |  | 0 | 1 |  |  | 0 | 1 | 0 | 4 |
| Week 12 | 0 | 2 |  |  |  |  |  |  | 0 | 1 | 0 | 3 |
| Week 52 | 0 | 1 |  |  | 0 | 1 |  |  | 0 | 1 | 0 | 3 |
| Insomnia |  |  |  |  |  |  |  |  |  |  |  |  |
| Hospitalization | 44 | 3 | 78 | 3 | 22 | 3 | 11 | 3 | 56 | 3 | 42 | 15 |
| Week 4 | 11 | 3 | 50 | 2 | 22 | 3 | 17 | 2 | 11 | 3 | 21 | 13 |
| Week 8 | 0 | 2 |  |  | 0 | 1 |  |  | 0 | 1 | 0 | 4 |
| Week 12 | 0 | 2 |  |  |  |  |  |  | 0 | 1 | 0 | 3 |
| Week 52 | 33 | 1 |  |  | 0 | 1 |  |  | 0 | 1 | 11 | 3 |
| Appetite loss |  |  |  |  |  |  |  |  |  |  |  |  |
| Hospitalization | 11 | 3 | 0 | 3 | 0 | 3 | 0 | 3 | 44 | 3 | 11 | 15 |
| Week 4 | 0 | 3 | 17 | 2 | 0 | 3 | 50 | 2 | 0 | 3 | 10 | 13 |
| Week 8 | 17 | 2 |  |  | 0 | 1 |  |  | 0 | 1 | 8 | 4 |
| Week 12 | 0 | 2 |  |  |  |  |  |  | 0 | 1 | 0 | 3 |
| Week 52 | 0 | 1 |  |  | 0 | 1 |  |  | 0 | 1 | 0 | 3 |
| Constipation |  |  |  |  |  |  |  |  |  |  |  |  |
| Hospitalization | 11 | 3 | 33 | 3 | 11 | 3 | 11 | 3 | 44 | 3 | 22 | 15 |
| Week 4 | 11 | 3 | 0 | 2 | 11 | 3 | 0 | 2 | 0 | 3 | 5 | 13 |
| Week 8 | 17 | 2 |  |  | 0 | 1 |  |  | 0 | 1 | 8 | 4 |
| Week 12 | 17 | 2 |  |  |  |  |  |  | 0 | 1 | 11 | 3 |
| Week 52 | 0 | 1 |  |  | 0 | 1 |  |  | 0 | 1 | 0 | 3 |
|  |  |  |  |  |  |  |  |  |  |  |  |  |
| Diarrhea |  |  |  |  |  |  |  |  |  |  |  |  |
| Hospitalization | 0 | 3 | 33 | 3 | 0 | 3 | 0 | 3 | 0 | 3 | 7 | 15 |
| Week 4 | 0 | 3 | 0 | 2 | 0 | 3 | 0 | 2 | 0 | 3 | 0 | 13 |
| Week 8 | 0 | 2 |  |  | 0 | 1 |  |  | 0 | 1 | 0 | 4 |
| Week 12 | 0 | 2 |  |  |  |  |  |  | 0 | 1 | 0 | 3 |
| Week 52 | 0 | 1 |  |  | 0 | 1 |  |  | 0 | 1 | 0 | 3 |
| Financial difficulties |  |  |  |  |  |  |  |  |  |  |  |  |
| Hospitalization | 11 | 3 | 33 | 3 | 22 | 3 | 0 | 3 | 22 | 3 | 18 | 15 |
| Week 4 | 11 | 3 | 50 | 2 | 11 | 3 | 0 | 2 | 0 | 3 | 13 | 13 |
| Week 8 | 17 | 2 |  |  | 67 | 1 |  |  | 33 | 1 | 33 | 4 |
| Week 12 | 17 | 2 |  |  |  |  |  |  | 33 | 1 | 22 | 3 |
| Week 52 | 0 | 1 |  |  | 67 | 1 |  |  | 67 | 1 | 44 | 3 |

**Supplementary Table S6: List of primers used for Sanger sequencing**

| ***Gene*** | **Mutation** | **Primer Forward** | **Primer Reverse** |
| --- | --- | --- | --- |
| *ATRX* | C.5375_5395del | TTTCAAAACGATATTCCAAGAG | ACTCCTATAATCATAATACATG |
| *BRAF* | c.G1465A | ACATTACTTGCAGTTTCAGTTAGT | GCTTGCTTCCCTGGTCTGCTCTCT |
| *CDKN2A* | c.G187C | AGCAGCATGGAGCCTTCGGCTGA | CAGCATTCGAGAGATCTGTACG |
| *EGFR* | c.C866T | CAGTAACTTGGGCTTTCTGA | AAGATGGGATACTCCAGGGC |
| *EGFR* | c.G1793T | GGTGCAATCACAGAATAACTGG | GTTGCCGGAAAACTTGGGAG |
| *ERBB2* | c.C3647A | AGCACGCAAGCTTTCTCCTGCTGT | CAGACATGACCTCGGCCAGCC |
| *IDH1* | c.G395A | ACCAAATGGCACCATACGA | TTCATACCTTGCTTAATGGGTGT |
| *MET* | c.C2962T | GTGCTACAACCTGTGTAGTAC | GAAATGAGAGCTTATGGACTC |
| *NF1* | c.C1591T | CGTCCAGCCTAGTTCTAGAACAT | GCAAGTAAACCCCTTCTTTCTC |
| *PIK3CA* | c.G1633A | ATCCAGAGGGGAAAAATATG | ATGCTGAGATCAGCCAAAT |
| *PTEN* | c.T593C | GTCAGAGCGCTGTTGTGACC | TATAATTTGGCTTCGACTAC |
| *PTEN* | c.955dupA | CTCAGATTGCCTTATAATAGT | CAAGTTCTTCATCAGCTGTACTC |
| *PTEN* | c.956_959del | CTCAGATTGCCTTATAATAGT | CAAGTTCTTCATCAGCTGTACTC |
| *TP53* | c.G830T | CCCCTGCTTGCCACAGGT | GTGAATCTGAGGCATAACTGC |
| *TP53* | c.A745G | CCCCTGCTTGCCACAGGT | GTGAATCTGAGGCATAACTGC |
| *TP53* | c.626_627del | GCCGTCTTCCAGTTGCTTTATCTG | TGGTGGTACAGTCAGAGCCAAC |
| *TP53* | c.G322A | CCTGGTCCTCTGACTGCTCTTTTCACCCA | GGCCAGGCATTGAAGTCTCAT |
