# Supplementary material for: Local delivery of hrBMP4 as an anticancer therapy in patients with recurrent glioblastoma: a first-in-human phase 1 dose escalation trial
Source: Mol Cancer. 2023 Aug 10;22:129. doi: 10.1186/s12943-023-01835-6 (PMC10413694; doi:10.1186/s12943-023-01835-6)
Supplement: Supplementary file 2 — Supplementary Material 2 [file 12943_2023_1835_MOESM2_ESM.pdf]

Pro-differentiation anticancer therapy using local delivery of hrBMP4 in patients with recurrent glioblastoma: a first-in-human Phase 1 dose escalation trial

Eelke M. Bos*[1], Elena Binda*[2], Iris S.C. Verploegh*[1,3], Eva Wembacher[4], Daphna Hoefnagel[1], Rutger K. Balvers[1], Anne L. Korporaal[2], Andrea Conidi[2], Esther A. H. Warnert[5], Nadia Trivieri[2], Alberto Visioli[11,12], Paola Zaccarini[11,12], Laura Caiola[11,12], Rogier van Wijck[6], Peter van der Spek[6], Danny Huylebroeck[2], Sieger Leenstra[1], Martine L.M. Lamfers[1], Zvi Ram[7], Manfred Westphal[8], David Noske[9], Federico Legnani[10], Francesco DiMeco[10], Angelo L. Vescovi[#§2,13], Clemens M.F. Dirven[§1]

[1]Department of Neurosurgery, Erasmus MC Cancer Institute, University Medical Center Rotterdam, The Netherlands

[2]Unit of Cancer Stem Cells, ISBReMIT, IRCCS Casa Sollievo della Sofferenza, San Giovanni Rotondo (FG), Italy

[3]Department of Cell Biology, Erasmus MC, University Medical Center Rotterdam, The Netherlands

[4]Brainlab A.G., Munich, Germany

[5]Department of Radiology, Erasmus MC Cancer Institute, University Medical Center Rotterdam, The Netherlands

[6]Department of Clinical Bioinformatics, Erasmus MC, University Medical Center Rotterdam, The Netherlands

[7]Department of Neurosurgery, Tel Aviv Medical Center, Israel

[8]Department of Neurosurgery, University Clinic Hamburg-Eppendorf, Hamburg, Germany

[9]Department of Neurosurgery, Amsterdam University Medical Center, The Netherlands

[10]Department of Neurosurgery, Istituto Neurologico C. Besta, Milan, Italy

[11] StemGen SpA, Milan, Italy

[12] HyperStem SA, Lugano, Switzerland

[13] Department of Biotechnology and Biosciences University of Milano-Bicocca, Milan, Italy

[#]**Corresponding author:**

Angelo Luigi Vescovi

IRCCS Casa Sollievo della Sofferenza, Scientific Directorate

Viale dei Cappuccini 1, 71013, San Giovanni Rotondo (FG), Italy

E-mail: angelo.vescovi@unimib.it

SUPPLEMENTARY MATERIALS

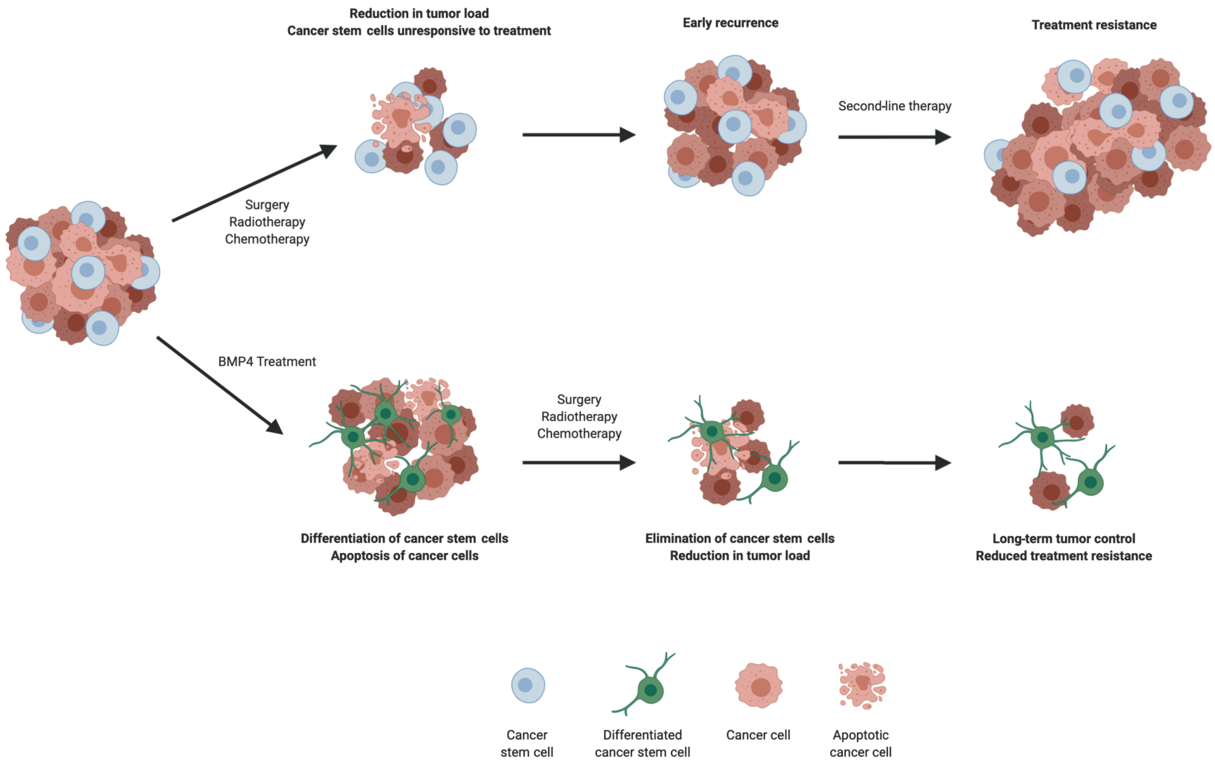

Supplementary Figure S1: Hypothetical schema of BMP4's mechanism of action as a PDT.

A

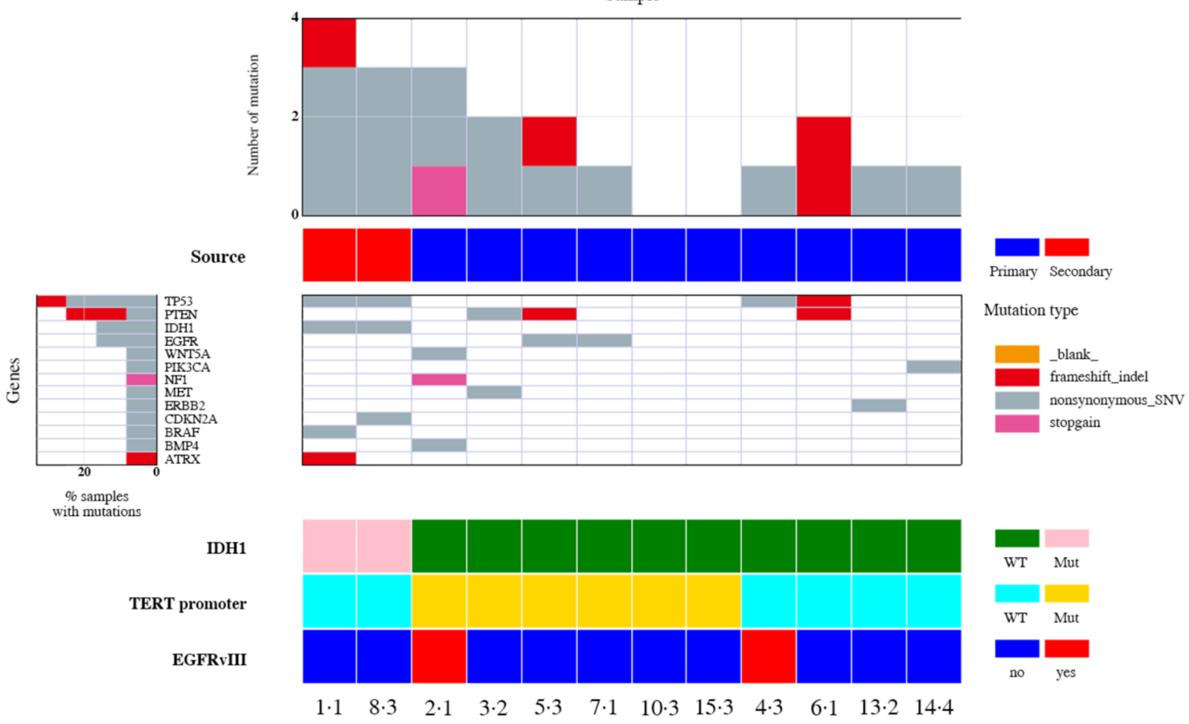

B

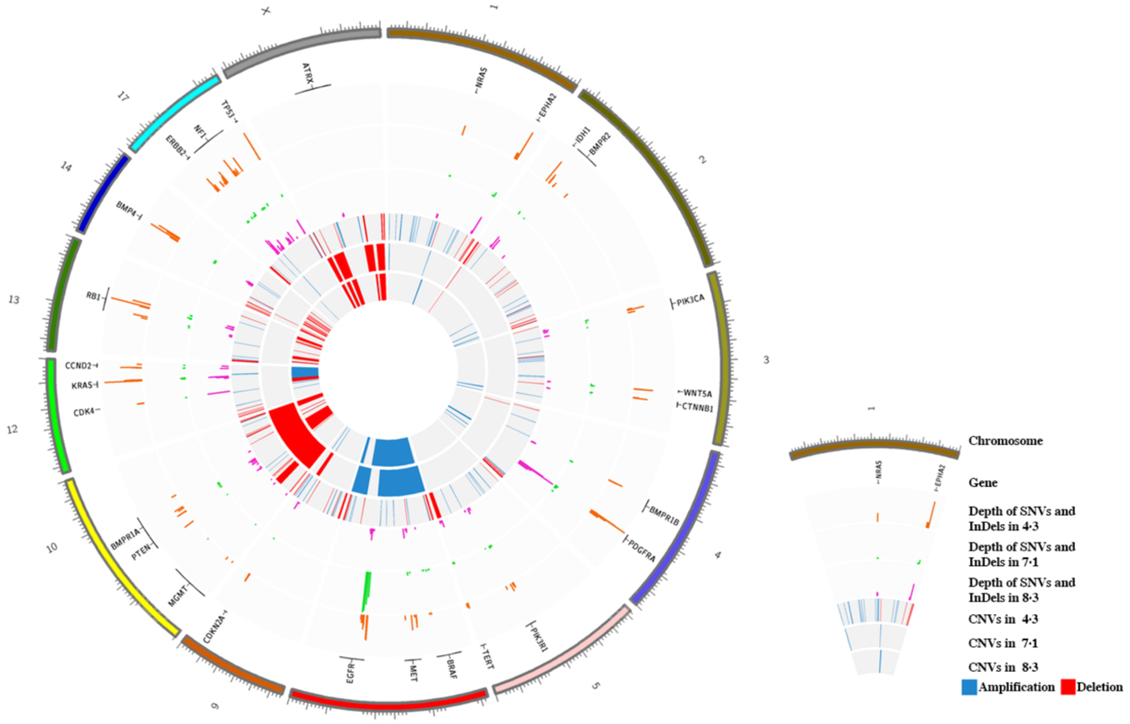

Supplementary Figure S2: Genetic analysis of tumors from patients included in this study. (A) Overview of mutations found in the treated patients. (B) Circos plot for genetic alterations detected in our samples. The outer track provides somatic single nucleotide variants (SNVs), small insertions, and deletions (indels); and the inner track CNVs. -log10 (q-value) of the significant amplified or deleted region in blue or red, respectively.

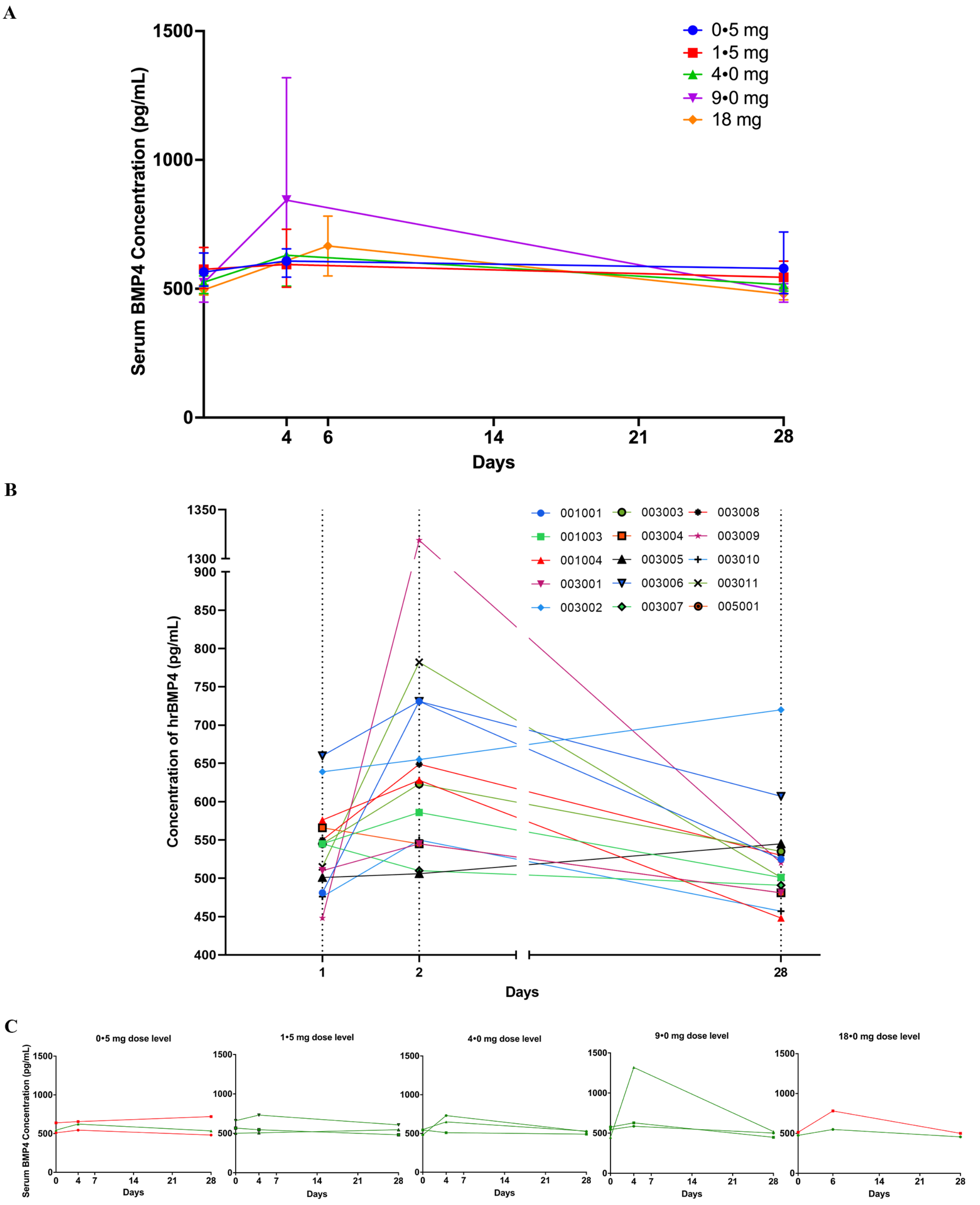

Supplementary Figure S3. (A) Mean serum levels of hrBMP4 in pg/ml at baseline (day 0), at the end of infusion with hrBMP4 (day 4 or 6, depending on dose level), and 4 weeks after infusion. Data are presented by dose level and displayed in different colors for each dose group. (B) Individual patient BMP4 systemic concentration profiles, showing serum levels of hrBMP4 (in pg/mL) at pre-infusion, the end of infusion, and 4 weeks after treatment. The majority of subjects experienced an increase in BMP4 systemic levels at the end of infusion, which returned

to baseline values at week 4. Both subjects who experienced Grade 3 lymphocyte count decrease presented an increase in BMP4 systemic concentration at the end of infusion which was the highest in subject 003009 while the increase measured for subject 003003 was superimposable to those observed in the other study participants. (C) Individual patient BMP4 systemic concentration profiles presented by dose level. Responder patients (003001, 003002 and 003011) are displayed in red whereas non responders patients are indicated by green lines.

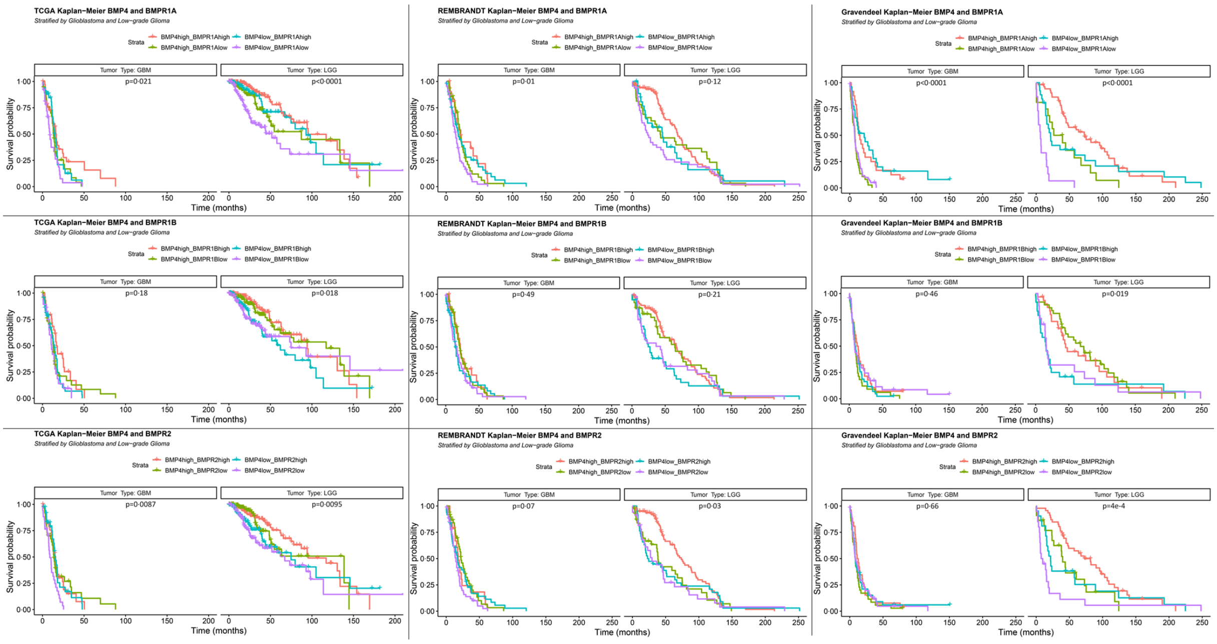

Supplementary Figure S4. Kaplan-Meier survival plots for low- and glioblastoma multiforme (LGG and GBM) in three independent public glioma datasets (TCGA, REMBRANDT and Gravendeel) showing that the increased expression of BMP4 and its BMPR1A receptor is associated with a better prognosis in low- and high-grade glioma patients.

SUPPLEMENTARY TABLES

Patient	Dose (mg)	Age	Gender	KPS	Survival before trial start (months)	IDH1 mutant	RT+TMZ (# TMZ cycles)*	Lomustine*	Re-resection*	Re-resection before hrBMP4	Tumor location
1	0·5	63	M	80	15	Y	Yes (6)				Left Temporal
2	0·5	56	F	80	22	N	Yes (6)	x	x		Right Temporal/Occipital
3	0·5	51	F	80	13	N	Yes (5)			x	Right Frontal/Temporal/Parietal
4	1·5	44	F	100	14	N	Yes (6)				Right Frontal
5	1·5	56	M	80	11	N	Yes (6)		x		Right Frontal
6	1·5	33	F	90	28	N	Yes (6)	x		x	Right Frontal
7	4	53	M	90	13	N	Yes (5)			x	Temporal/Parietal/Occipital
8	4	38	M	90	33	Y	Yes (2)				Left Frontal/Temporal/Parietal
9	4	40	M	100	14		Yes (11)			x	
10	9	56	M	90	11	N	Yes (5)				Right Temporal
11	9	56	M	90	26		Yes (21)			x	
12	9	64	M	80	8		Yes (0)			x	
13	18	36	M	90	8	N	Yes (2)			x	Left Frontal/Parietal
14	18	70	F	80	26	N	Yes (6)				Left Temporal/Occipital
15	18	63	M	90	14	N	Yes (5)	x			Left Parietal

*Before inclusion into this trial

Supplementary Table S1: Patient demographic characteristics

Patient	BMP4 Dose (mg)	GBM Subgroup	IDH1 mutant	TERT promoter mutation	EGFRvIII mutation	1p19q codeletion	CHR7 amplification	CHR10 deletion	Mutated Genes in NGS Panel
1	0·5	Secondary IDH1^{MUT}-TERTp^{WT}	Y	WT	NO	NO	NO	NO	BRAF, TP53, ATRX
2	0·5	Primary IDH1^{WT}-TERTp^{MUT}	N	C228T	YES	NO	YES	YES	WNT5A, BMP4, TP53
3	0·5	Primary IDH1^{WT}-TERTp^{MUT}	N	C228T	NO	NO	YES	YES	MET, PTEN
4	1·5	Primary IDH1^{WT}-TERTp^{WT}	N	WT	YES	NO	YES	YES	TP53
5	1·5	Primary IDH1^{WT}-TERTp^{MUT}	N	C288T	NO	NO	YES	YES	EGFR, PTEN
6	1·5	Primary IDH1^{WT}-TERTp^{WT}	N	WT	NO	NO	YES	NO	PTEN, TP53
7	4	Primary IDH1^{WT}-TERTp^{MUT}	N	C288T	NO	NO	YES	YES	EGFR
8	4	Secondary IDH1^{MUT}-TERTp^{WT}	Y	WT	NO	NO	YES	YES	CDKN2A, TP53
9	4								
10	9	Primary IDH1^{WT}-TERTp^{MUT}	N	C250T	NO	NO	YES	YES	
11	9								
12	9								
13	18	Primary IDH1^{WT}-TERTp^{WT}	N	WT	NO	NO	NO	NO	ERBB2
14	18	Primary IDH1^{WT}-TERTp^{WT}	N	WT	NO	NO	YES	YES	PIK3CA
15	18	Primary IDH1^{WT}-TERTp^{MUT}	N	C288T	NO	NO	YES	YES	

Supplementary Table S2: Tumor genetic characteristics

Supplementary Table S3: Overview of reported adverse events

	**Number of events**					
Event	0·5 mg (n=3)	1·5 mg (n=3)	4·0 mg (n=3)	9·0 mg (n=3)	18 mg (n=3)	Total (n=15)
AEs	19	13	20	17	28	97
Related	1	0	0	1	6	8
SAEs	3	3	4	3	4	17
Related	0	0	0	0	0	0
DLT	0	0	0	0	0	0

Supplementary Table S4: Summary of Treatment Emergent Adverse Events (TEAE) by System Organ Class (in ≥ 25% of patients) and Preferred Term

Overall	hrBMP4 0·5 mg (N=3)		hrBMP4 1·5 mg (N=3)		hrBMP4 4 mg (N=3)		hrBMP4 9 mg (N=3)		hrBMP4 18 mg (N=3)		Overall (N=15)	
	E	n	E	n	E	n	E	n	E	n	E	n (%)
**Any TEAE**	19	3	13	3	20	3	17	3	28	3	97	15 (100·0%)
**Blood and lymphatic system disorders**	1	1	-		-		3	2	1	1	5	4 (26·7%)
Anaemia	1	1	-		-		1	1	1	1	3	3 (20·0%)
Leukopenia	-		-		-		1	1	-		1	1 (6·7%)
Lymphopenia	-		-		-		1	1	-		1	1 (6·7%)
**Gastrointestinal disorders**	2	1	-		1	1	1	1	4	3	8	6 (40·0%)
Gastritis	-		-		-		1	1	-		1	1 (6·7%)
Nausea	1	1	-		-		-		1	1	2	2 (13·3%)
Vomiting	1	1	-		1	1	-		3	3	5	5 (33·3%)
**General disorders and administration site conditions**	1	1	-		3	2	-		2	1	6	4 (26·7%)
Catheter site haemorrhage	-		-		1	1	-		-		1	1 (6·7%)
Euthanasia	1	1	-		1	1	-		-		2	2 (13·3%)
Pain	-		-		-		-		1	1	1	1 (6·7%)
Pyrexia	-		-		1	1	-		1	1	2	2 (13·3%)
**Injury, poisoning and procedural complications**	1	1	-		1	1	-		2	2	4	4 (26·7%)
Wound	1	1	-		-		-		-		1	1 (6·7%)
Wound complication	-		-		1	1	-		1	1	2	2 (13·3%)
Wound infection	-		-		-		-		1	1	1	1 (6·7%)
**Investigations**	5	2	2	1	6	2	2	1	5	2	20	8 (53·3%)
Alanine aminotransferase increased	1	1	-		-		-		1	1	2	2 (13·3%)
Aspartate aminotransferase increased	1	1	-		1	1	-		1	1	3	3 (20·0%)
Blood bicarbonate increased	-		-		1	1	-		1	1	2	2 (13·3%)
Blood bilirubin increased	-		-		1	1	-		-		1	1 (6·7%)
Blood creatine decreased	-		-		-		-		1	1	1	1 (6·7%)
Blood creatinine increased	1	1	-		-		-		-		1	1 (6·7%)
Blood lactate dehydrogenase increased	-		1	1	1	1	1	1	-		3	3 (20·0%)
Gamma-glutamyltransferase increased	1	1	-		1	1	-		-		2	2 (13·3%)
Lymphocyte count decreased	1	1	-		1	1	1	1	1	1	4	4 (26·7%)
Red blood cell count decreased	-		1	1	-		-		-		1	1 (6·7%)
**Metabolism and nutrition disorders**	-		2	1	2	2	1	1	4	2	9	6 (40·0%)
Hyperglycaemia	-		1	1	2	2	-		1	1	4	4 (26·7%)
Hypoalbuminaemia	-		-		-		-		1	1	1	1 (6·7%)
Hypokalaemia	-		-		-		1	1	-		1	1 (6·7%)

Overall	hrBMP4 0·5 mg (N=3)		hrBMP4 1·5 mg (N=3)		hrBMP4 4 mg (N=3)		hrBMP4 9 mg (N=3)		hrBMP4 18 mg (N=3)		Overall (N=15)	
	E	n	E	n	E	n	E	n	E	n	E	n (%)
Hypophosphataemia	-		1	1	-		-		2	2	3	3 (20·0%)
**Neoplasms benign, malignant and unspecified (incl cysts and polyps)**	2	2	3	3	3	3	2	2	3	2	13	12 (80·0%)
Neoplasm progression	2	2	3	3	3	3	2	2	3	2	13	12 (80·0%)
**Nervous system disorders**	7	2	5	2	4	2	4	2	3	2	23	10 (66·7%)
Aphasia	1	1	-		-		-		-		1	1 (6·7%)
Extensor plantar response	1	1	-		-		-		-		1	1 (6·7%)
Headache	1	1	2	2	2	2	4	2	2	2	11	9 (60·0%)
Hemiparesis	2	2	1	1	2	1	-		-		5	4 (26·7%)
Muscle spasticity	1	1	-		-		-		-		1	1 (6·7%)
Nervous system disorders	-		1	1	-		-		-		1	1 (6·7%)
Seizure	-		-		-		-		1	1	1	1 (6·7%)
Sensory loss	1	1	-		-		-		-		1	1 (6·7%)
Tremor	-		1	1	-		-		-		1	1 (6·7%)

Supplementary Table S5: Overview of Quality-of-Life reporting

EORTC QLQ-C30	0·5 mg		1·5 mg		4·0 mg		9·0 mg		18·0 mg		Total	
	Mean	n	Mean	n	Mean	n	Mean	n	Mean	n	Mean	n
**Functioning scales**												
Physical												
Hospitalization	71	3	62	3	87	3	84	3	89	3	79	15
Week 4	29	3	60	2	89	3	70	2	84	3	67	13
Week 8	57	2			80	1			100	1	73	4
Week 12	43	2							100	1	62	3
Week 52	93	1			53	1			100	1	82	3
Role												
Hospitalization	39	3	22	3	56	3	83	3	61	3	52	15
Week 4	22	3	50	2	56	3	58	2	78	3	53	13
Week 8	33	2			33	1			100	1	50	4
Week 12	25	2							100	1	50	3
Week 52	33	1			33	1			67	1	44	3
Emotional												
Hospitalization	72	3	83	2	78	3	83	3	69	3	77	14
Week 4	67	3	38	2	61	3	71	2	81	3	65	13
Week 8	96	2			75	1			100	1	92	4
Week 12	63	2							100	1	75	3
Week 52	50	1			67	1			100	1	72	3
Cognitive												
Hospitalization	61	3	44	3	83	3	72	3	67	3	66	15
Week 4	50	3	33	2	61	3	67	2	67	3	56	13
Week 8	50	2			100	1			83	1	71	4
Week 12	33	2							83	1	50	3
Week 52	67	1			100	1			83	1	83	3
Social												
Hospitalization	56	3	50	3	94	3	78	3	89	3	73	15
Week 4	56	3	33	2	78	3	58	2	94	3	67	13
Week 8	33	2			83	1			100	1	63	4
Week 12	33	2							100	1	56	3
Week 52	50	1			83	1			83	1	72	3
Global												
Hospitalization	50	3	69	3	75	3	72	3	50	3	63	15
Week 4	36	3	58	2	61	3	58	2	67	3	56	13
Week 8	54	2			33	1			62	1	58	4
Week 12	50	2							83	1	61	3
Week 52	83	1			33	1			83	1	67	3

**Symptom scales**												
Fatigue												
Hospitalization	37	3	61	2	41	3	26	3	52	3	42	14
Week 4	63	3	72	2	37	3	50	2	30	3	49	13
Week 8	56	2			22	1			11	1	36	4
Week 12	50	2							0	1	33	3
Week 52	22	1			56	1			0	1	26	3
Nausea/vomiting												
Hospitalization	6	3	11	3	0	3	0	3	56	3	14	15
Week 4	6	3	8	2	11	3	0	2	0	3	5	13
Week 8	0	2			0	1			0	1	0	4
Week 12	0	2							0	1	0	3
Week 52	0	1			0	1			0	1	0	3
Pain												
Hospitalization	22	3	39	3	28	3	6	3	22	3	23	15
Week 4	33	3	25	2	28	3	8	2	11	3	22	13
Week 8	0	2			0	1			0	1	0	4
Week 12	0	2							0	1	0	3
Week 52	0	1			0	1			0	1	0	3
Dyspnea												
Hospitalization	22	3	44	3	0	3	0	3	11	3	16	15
Week 4	22	3	0	2	0	3	33	2	0	3	10	13
Week 8	0	2			0	1			0	1	0	4
Week 12	0	2							0	1	0	3
Week 52	0	1			0	1			0	1	0	3
Insomnia												
Hospitalization	44	3	78	3	22	3	11	3	56	3	42	15
Week 4	11	3	50	2	22	3	17	2	11	3	21	13
Week 8	0	2			0	1			0	1	0	4
Week 12	0	2							0	1	0	3
Week 52	33	1			0	1			0	1	11	3
Appetite loss												
Hospitalization	11	3	0	3	0	3	0	3	44	3	11	15
Week 4	0	3	17	2	0	3	50	2	0	3	10	13
Week 8	17	2			0	1			0	1	8	4
Week 12	0	2							0	1	0	3
Week 52	0	1			0	1			0	1	0	3
Constipation												
Hospitalization	11	3	33	3	11	3	11	3	44	3	22	15
Week 4	11	3	0	2	11	3	0	2	0	3	5	13
Week 8	17	2			0	1			0	1	8	4
Week 12	17	2							0	1	11	3

Week 52	0	1			0	1			0	1	0	3
Diarrhea												
Hospitalization	0	3	33	3	0	3	0	3	0	3	7	15
Week 4	0	3	0	2	0	3	0	2	0	3	0	13
Week 8	0	2			0	1			0	1	0	4
Week 12	0	2							0	1	0	3
Week 52	0	1			0	1			0	1	0	3
Financial difficulties												
Hospitalization	11	3	33	3	22	3	0	3	22	3	18	15
Week 4	11	3	50	2	11	3	0	2	0	3	13	13
Week 8	17	2			67	1			33	1	33	4
Week 12	17	2							33	1	22	3
Week 52	0	1			67	1			67	1	44	3

Supplementary Table S6: List of primers used for Sanger sequencing

Gene	Mutation	Primer Forward	Primer Reverse
ATRX	C.5375_5395del	TTTCAAAACGATATTCCAAGAG	ACTCCTATAATCATAATACATG
BRAF	c.G1465A	ACATTACTTGCAGTTTCAGTTAGT	GCTTGCTTCCCTGGTCTGCTCTCT
CDKN2A	c.G187C	AGCAGCATGGAGCCTTCGGCTGA	CAGCATTCGAGAGATCTGTACG
EGFR	c.C866T	CAGTAACTTGGGCTTTCTGA	AAGATGGGATACTCCAGGGC
EGFR	c.G1793T	GGTGCAATCACAGAATAACTGG	GTTGCCGGAAAACTTGGGAG
ERBB2	c.C3647A	AGCACGCAAGCTTTCTCCTGCTGT	CAGACATGACCTCGGCCAGCC
IDH1	c.G395A	ACCAAATGGCACCATACGA	TTCATACCTTGCTTAATGGGTGT
MET	c.C2962T	GTGCTACAACCTGTGTAGTAC	GAAATGAGAGCTTATGGACTC
NF1	c.C1591T	CGTCCAGCCTAGTTCTAGAACAT	GCAAGTAAACCCCTTCTTTCTC
PIK3CA	c.G1633A	ATCCAGAGGGGAAAAATATG	ATGCTGAGATCAGCCAAAT
PTEN	c.T593C	GTCAGAGCGCTGTTGTGACC	TATAATTTGGCTTCGACTAC
PTEN	c.955dupA	CTCAGATTGCCTTATAATAGT	CAAGTTCTTCATCAGCTGTACTC
PTEN	c.956_959del	CTCAGATTGCCTTATAATAGT	CAAGTTCTTCATCAGCTGTACTC
TP53	c.G830T	CCCCTGCTTGCCACAGGT	GTGAATCTGAGGCATAACTGC
TP53	c.A745G	CCCCTGCTTGCCACAGGT	GTGAATCTGAGGCATAACTGC
TP53	c.626_627del	GCCGTCTTCCAGTTGCTTTATCTG	TGGTGGTACAGTCAGAGCCAAC
TP53	c.G322A	CCTGGTCCTCTGACTGCTCTTTTCACCCA	GGCCAGGCATTGAAGTCTCAT
