# Supplementary material for: Local delivery of hrBMP4 as an anticancer therapy in patients with recurrent glioblastoma: a first-in-human phase 1 dose escalation trial
Source: Mol Cancer. 2023 Aug 10;22:129. doi: 10.1186/s12943-023-01835-6 (PMC10413694; doi:10.1186/s12943-023-01835-6)
Supplement: Supplementary file 3 — Supplementary Material 3 [file 12943_2023_1835_MOESM3_ESM.pdf]

Pro-differentiation anticancer therapy using local delivery of hrBMP4 in patients with recurrent glioblastoma: a first-in-human Phase 1 dose escalation trial

Eelke M. Bos*[1], Elena Binda*[2], Iris S.C. Verploegh*[1,3], Eva Wembacher[4], Daphna Hoefnagel[1], Rutger K. Balvers[1], Anne L. Korporaal[3], Andrea Conidi[3], Esther A. H. Warnert[5], Nadia Trivieri[2], Alberto Visioli[11,12], Paola Zaccarini[11,12], Laura Caiola[11,12], Rogier van Wijck[6], Peter van der Spek[6], Danny Huylebroeck[3], Sieger Leenstra[1], Martine L.M. Lamfers[1], Zvi Ram[7], Manfred Westphal[8], David Noske[9], Federico Legnani[10], Francesco DiMeco[10], Angelo L. Vescovi[#§2,13], Clemens M.F. Dirven[§1]

[1]Department of Neurosurgery, Erasmus MC Cancer Institute, University Medical Center Rotterdam, The Netherlands

[2]Unit of Cancer Stem Cells, ISBReMIT, IRCCS Casa Sollievo della Sofferenza, San Giovanni Rotondo (FG), Italy

[3]Department of Cell Biology, Erasmus MC, University Medical Center Rotterdam, The Netherlands

[4]Brainlab A.G., Munich, Germany

[5]Department of Radiology, Erasmus MC Cancer Institute, University Medical Center Rotterdam, The Netherlands

[6]Department of Clinical Bioinformatics, Erasmus MC, University Medical Center Rotterdam, The Netherlands

[7]Department of Neurosurgery, Tel Aviv Medical Center, Israel

[8]Department of Neurosurgery, University Clinic Hamburg-Eppendorf, Hamburg, Germany

[9]Department of Neurosurgery, Amsterdam University Medical Center, The Netherlands

[10]Department of Neurosurgery, Istituto Neurologico C. Besta, Milan, Italy

[11] StemGen SpA, Milan, Italy

[12] HyperStem SA, Lugano, Switzerland

[13] Department of Biotechnology and Biosciences University of Milano-Bicocca, Milan, Italy

[#]**Corresponding author:**

Angelo Luigi Vescovi

IRCCS Casa Sollievo della Sofferenza, Scientific Directorate

Viale dei Cappuccini 1, 71013, San Giovanni Rotondo (FG), Italy

E-mail: angelo.vescovi@unimib.it

SUPPLEMENTARY MATERIALS

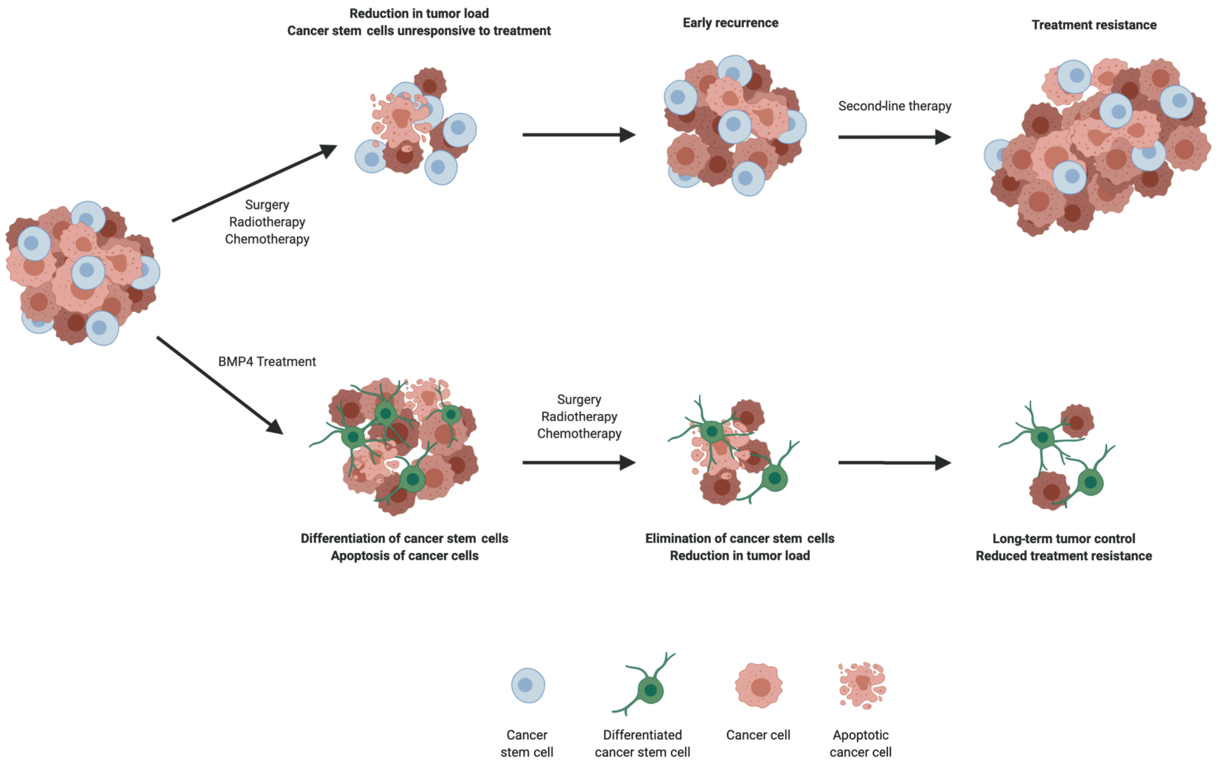

Supplementary Figure S1: Hypothetical schema of BMP4's mechanism of action as a PDT.

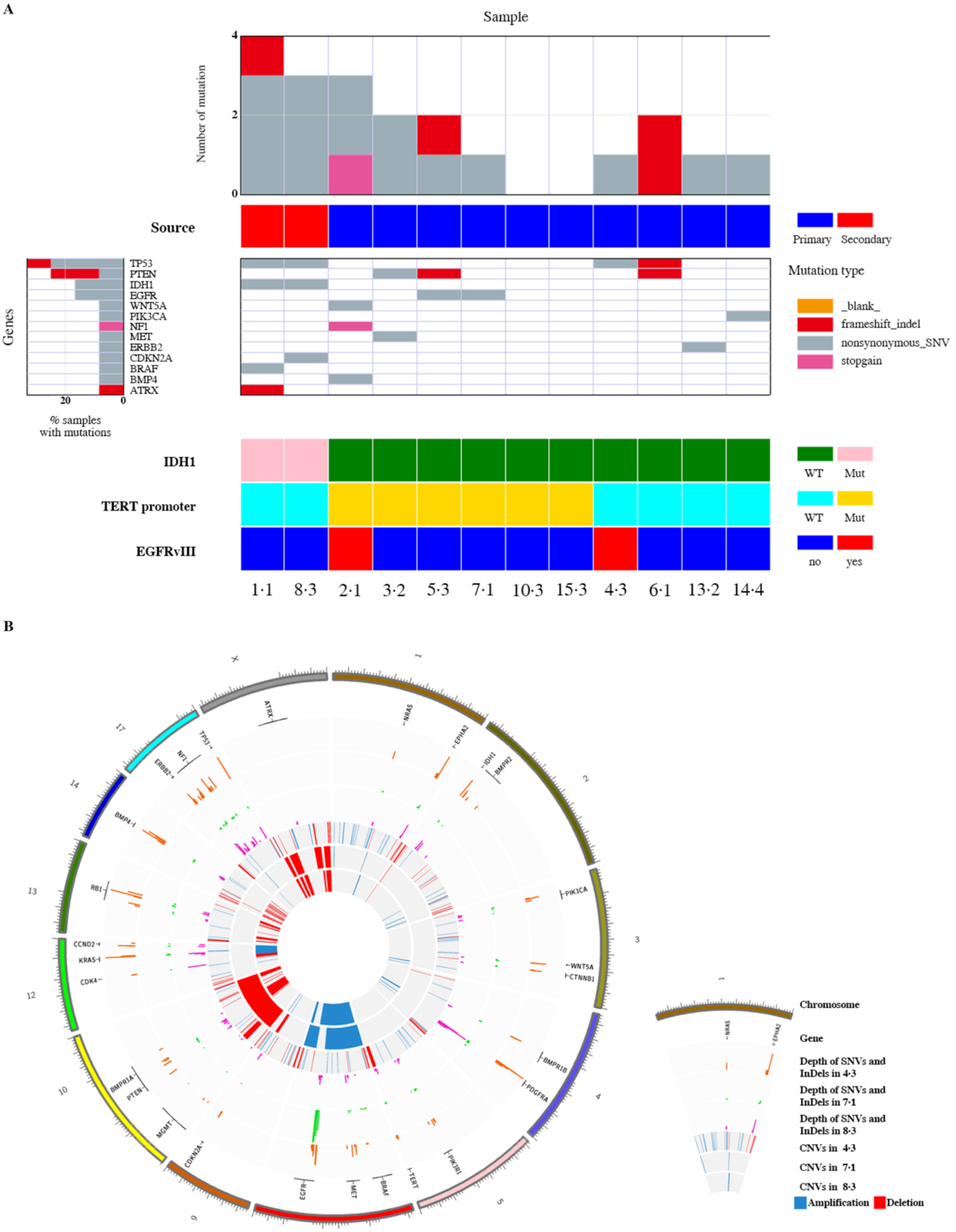

Supplementary Figure S2: Genetic analysis of tumors from patients included in this study. (A) Overview of mutations found in the treated patients. (B) Circos plot for genetic alterations detected in our samples. The outer track provides somatic single nucleotide variants (SNVs), small insertions, and deletions (indels); and the inner track CNVs. -log10 (q-value) of the significant amplified or deleted region in blue or red, respectively (see Supplementary Table7).

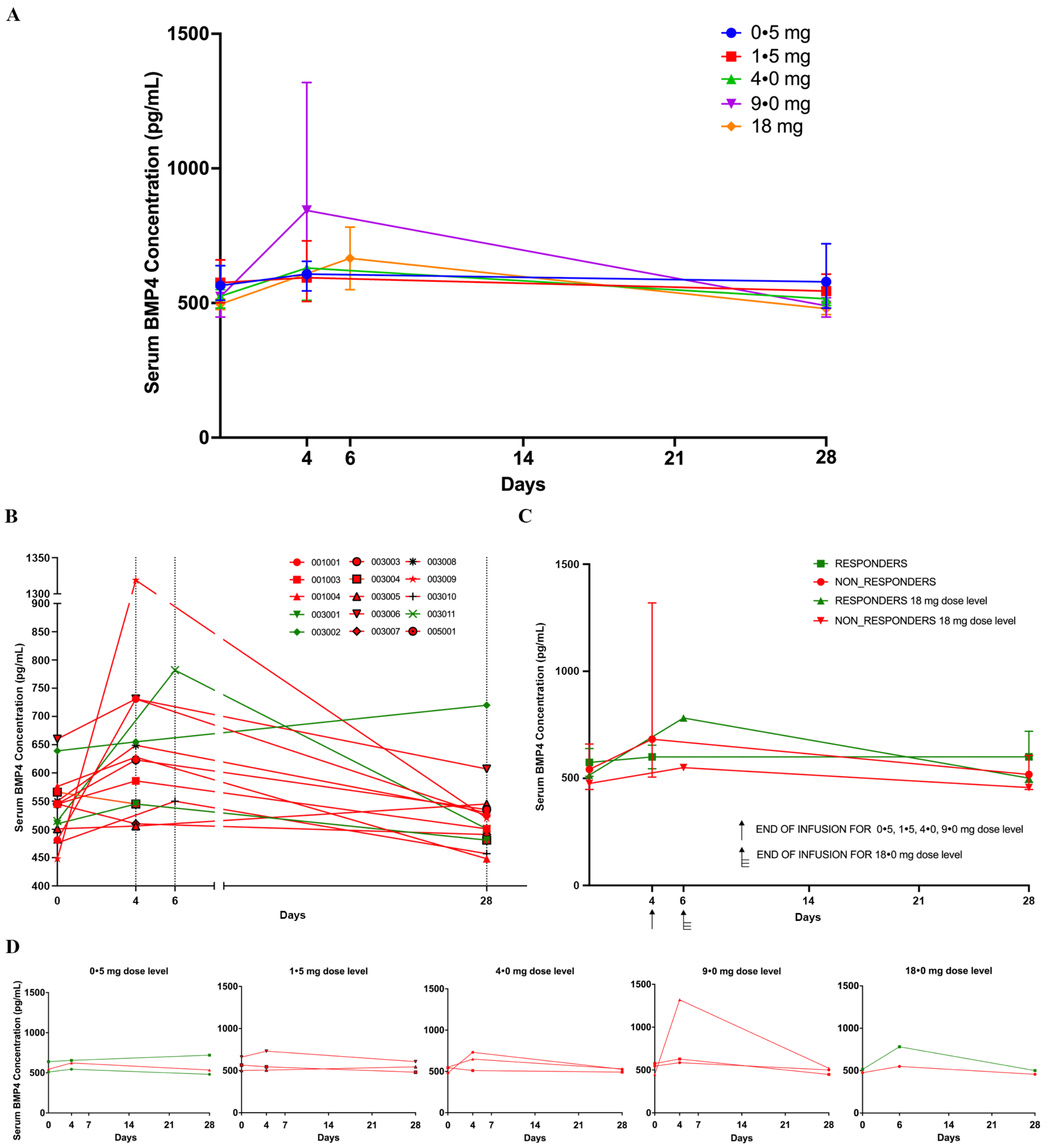

Supplementary Figure S3. (A) Mean serum levels of hrBMP4 in pg/ml at baseline (day 0), at the end of infusion with hrBMP4 (day 4 or 6, depending on dose level), and 4 weeks after infusion. Data are presented by dose level and displayed in different colors for each dose group. (B) Individual patient BMP4 systemic concentration profiles, showing serum levels of hrBMP4 (in pg/mL) at pre-infusion, the end of infusion, and 4 weeks after treatment. The majority of subjects experienced an increase in BMP4 systemic levels at the end of infusion, which returned to baseline values at week 4. Both subjects who experienced Grade 3 lymphocyte count decrease presented an increase in BMP4 systemic concentration at the end of infusion which

was the highest in subject 003009 while the increase measured for subject 003003 was superimposable to those observed in the other study participants. Responders patients (003001, 003002 and 003011) are displayed in green whereas non Responders patients are indicated by red lines. (C) Mean (with Full Range) BMP4 circulating levels of Responders (depicted in green) versus Non responders (in red) at baseline (Pre-Infusion), End of Infusion and at Week 4 from the start of infusion. Two separate concentration profiles are presented for both Responders and non Responders depending on the duration of infusion (4 days for the lower dose groups and 6 days for the 18.0 mg dose). The concentration profiles appear similar with a greater variability at the end of infusion in the non Responders for the presence of an outlier in this patient subset. (D) Individual patient BMP4 systemic concentration profiles presented by dose level. Green and red lines, responders and non Responders patients, respectively.

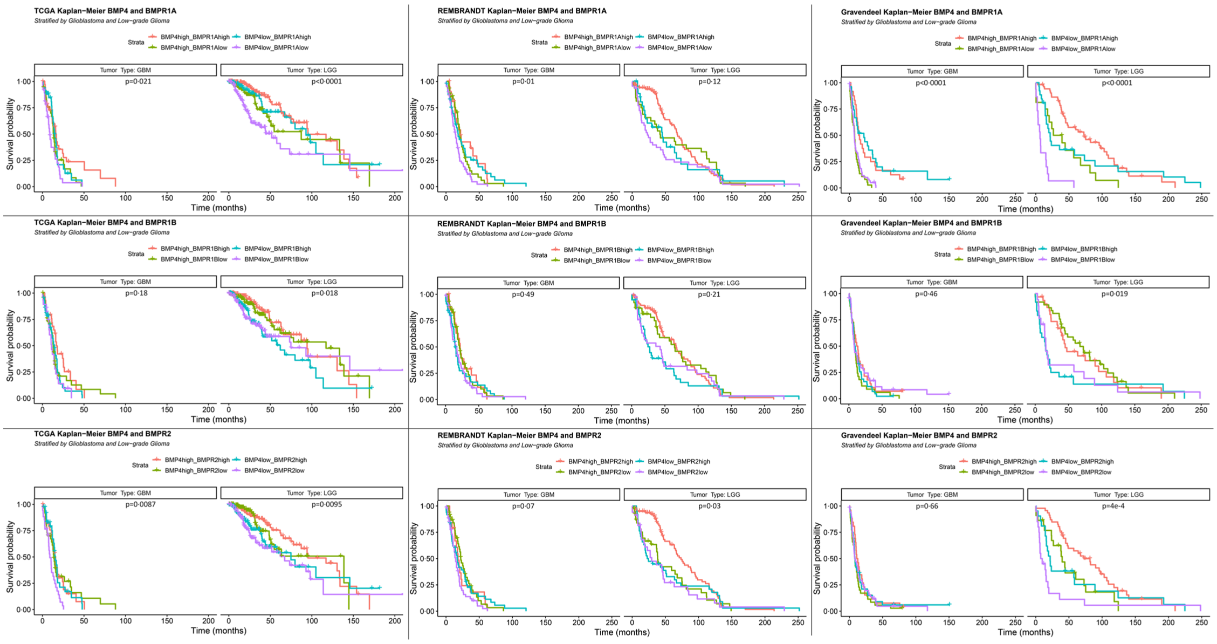

Supplementary Figure S4. Kaplan-Meier survival plots for low- and glioblastoma multiforme (LGG and GBM) in three independent public glioma datasets (TCGA, REMBRANDT and Gravendeel) showing that the increased expression of BMP4 and its BMPR1A receptor is associated with a better prognosis in low- and high-grade glioma patients.

SUPPLEMENTARY TABLES

Supplementary Table S1: Patient demographic characteristics

Patient	Dose (mg)	Age	Gender	KPS	Survival before trial start (months)	IDH1 mutant	RT+TMZ (# TMZ cycles)*	Lomustine*	Re-resection*	Re-resection before hrBMP4	Tumor location
1	0·5	63	M	80	15	Y	Yes (6)				Left Temporal
2	0·5	56	F	80	22	N	Yes (6)	x	x		Right Temporal/Occipital
3	0·5	51	F	80	13	N	Yes (5)			x	Right Frontal/Temporal/Parietal
4	1·5	44	F	100	14	N	Yes (6)				Right Frontal
5	1·5	56	M	80	11	N	Yes (6)		x		Right Frontal
6	1·5	33	F	90	28	N	Yes (6)	x		x	Right Frontal
7	4	53	M	90	13	N	Yes (5)			x	Temporal/Parietal/Occipital
8	4	38	M	90	33	Y	Yes (2)				Left Frontal/Temporal/Parietal
9	4	40	M	100	14		Yes (11)			x	
10	9	56	M	90	11	N	Yes (5)				Right Temporal
11	9	56	M	90	26		Yes (21)			x	
12	9	64	M	80	8		Yes (0)			x	
13	18	36	M	90	8	N	Yes (2)			x	Left Frontal/Parietal
14	18	70	F	80	26	N	Yes (6)				Left Temporal/Occipital
15	18	63	M	90	14	N	Yes (5)	x			Left Parietal

*Before inclusion into this trial

Supplementary Table S2: Tumor genetic characteristics

Patient	BMP4 Dose (mg)	GBM Subgroup	IDH1 mutant	TERT promoter mutation	EGFRvIII mutation	1p19q codeletion	CHR7 amplification	CHR10 deletion	Mutated Genes in NGS Panel
1	0·5	Secondary IDH1^{MUT}-TERTp^{WT}	Y	WT	NO	NO	NO	NO	BRAF, TP53, ATRX
2	0·5	Primary IDH1^{WT}-TERTp^{MUT}	N	C228T	YES	NO	YES	YES	WNT5A, BMP4, TP53
3	0·5	Primary IDH1^{WT}-TERTp^{MUT}	N	C228T	NO	NO	YES	YES	MET, PTEN
4	1·5	Primary IDH1^{WT}-TERTp^{WT}	N	WT	YES	NO	YES	YES	TP53
5	1·5	Primary IDH1^{WT}-TERTp^{MUT}	N	C288T	NO	NO	YES	YES	EGFR, PTEN
6	1·5	Primary IDH1^{WT}-TERTp^{WT}	N	WT	NO	NO	YES	NO	PTEN, TP53
7	4	Primary IDH1^{WT}-TERTp^{MUT}	N	C288T	NO	NO	YES	YES	EGFR
8	4	Secondary IDH1^{MUT}-TERTp^{WT}	Y	WT	NO	NO	YES	YES	CDKN2A, TP53
9	4								
10	9	Primary IDH1^{WT}-TERTp^{MUT}	N	C250T	NO	NO	YES	YES	
11	9								
12	9								
13	18	Primary IDH1^{WT}-TERTp^{WT}	N	WT	NO	NO	NO	NO	ERBB2
14	18	Primary IDH1^{WT}-TERTp^{WT}	N	WT	NO	NO	YES	YES	PIK3CA
15	18	Primary IDH1^{WT}-TERTp^{MUT}	N	C288T	NO	NO	YES	YES	

Supplementary Table S3: BMP4 Systemic Levels (pg/mL) in Selected Subgroups

A-Responders

Patient N°	BMP4 Dose group	Day 1 Pre-Infusion 18 Jul 17	Day 2	Day 3	Day 4	Day 5 End of infusion 22 Jul 17	Day 6	Week 4 25 Aug 17	Tumor Resection	Tumor Response*
003001	0.5 mg	510				545		481	Yes	PR 11 Oct 2017 (Week12); PD 09 Jan 18 (Week 24)
		Day 1 Pre-Infusion 29 Aug 17	Day 2	Day 3	Day 4	Day 5 End of infusion 02 Sep 17		Week 4 28 Sep 17		
003002	0.5 mg	639				655		720	No	PR from 22 Nov 17 (week 12) to 09 May 18 (Week 36); CR 15 Aug 18 (Week 48)
		Day 1 Pre-Infusion 16 Oct 19	Day 2	Day 3	Day 4	Day 5	Day 6 End of Infusion 22 Oct 19	Week 4 12 Nov 19		
003011	18.0 mg	515					782	501	No	PR from 22 Apr 2020 (week 24) to 12Oct 2019 (Week 48); CR Jan 2021 (Week 72)

*McDonald Criteria for GBM: CR= Complete Response; PR= Partial Response; SD= Stable Disease; PD= Progressive Disease

B-Non-Responders

Patient N°	BMP4 Dose group	Day 1 Pre-Infusion 10 Oct 17	Day 2	Day 3	Day 4	Day 5 End of infusion 14 Oct 17	Day 6	Week 4 08 Nov 17	Tumor Resection	Tumor Response
003003	0.5 mg	545				623		535	Yes	PD 08 Nov18 (Week 4)
		Day 1 Pre-Infusion 28 Nov 17	Day 2	Day 3	Day 4	Day 5 End of infusion 02 Dec 17		Week 4 10 Jan 18		
003004	1.5 mg	566				545		481	No	PD 10 Jan 18 (week 4)
		Day 1 Pre-Infusion 20 Dec 17	Day 2	Day 3	Day 4	Day 5 End of infusion 24 Dec 17		Week 4 26 Jan 18		
003005	1.5 mg	501				506		545	No	PD 09 Jan 18 (Week 4)
		Day 1 Pre-Infusion 06 Mar 18	Day 2	Day 3	Day 4	Day 5 End of infusion 10 Mar 18		Week 4 05 Apr 18		
003006	1.5 mg	660				731		607	Yes	PD 05 Apr 18 (week 4)
		Day 1 Pre-Infusion 23 Jul 18	Day 2	Day 3	Day 4	Day 5 End of infusion 27 Jul 18		Week 4 29 Aug 18		
001001	4.0 mg	545				586		501	Yes	SD 29 Aug 18 (Week 4) and 26 Sep 18 (week 8) PD 09 Oct 18 (Week 10)
		Day 1 Pre-Infusion 28 May 18	Day 2	Day 3	Day 4	Day 5 End of infusion 01 Jun 18		Week 4 26 Jun 18		
003007	4.0 mg	545				510		491	Yes	PD 26 Jun 18 (Week 4)
		Day 1 Pre-Infusion 16 Jul 18	Day 2	Day 3	Day 4	Day 5 End of infusion 20 Jul 18		Week 4 14 Aug 18		
003008	4.0 mg	550				649		530	No	PD 14 Aug 18(week 4)
		Day 1 Pre-Infusion 19 Nov 18	Day 2	Day 3	Day 4	Day 5 End of infusion		Week 4 20 Dec 18		

						23 Nov18				
001003	9.0 mg	545				586		501	Yes	PD 20 Dec 18 (Week 4)
		Day 1 Pre-Infusion 17 Dec 18	Day 2	Day 3	Day 4	Day 5 End of infusion 21 Dec 18		Week 4		
001004	9.0 mg	576				628		Not Done	Yes	Not Available (Consent withdrawal)
		Day 1 Pre-Infusion 15 Oct 18	Day 2	Day 3	Day 4	Day 5 End of infusion 19 Oct 18		Week 4 09 Nov 18		
003009	9.0 mg	448				1319		520	No	PD 09 Nov 18 (Week 4)
		Day 1 Pre-Infusion 26 Mar 19	Day 2	Day 3	Day 4	Day 5 End of infusion 01 Apr 19		Week 4 30 Apr 19		
003010	18.0 mg	476				550		459	Yes	PD 30 Apr 19 (Week 4)
		Day 1 Pre-Infusion 02 Apr 19	Day 2	Day 3	Day 4	Day 5	Day 6 End of Infusion 08 Apr 19	Week 4 13 May 2019		
005011	18.0 mg	Not Done					Not Done	Not Done	No	PD 08 May 18 (Week 4)
*McDonald Criteria for GBM: CR= Complete Response; PR= Partial Response; SD= Stable Disease; PD= Progressive Disease										

C- Overview of Subjects with Grade 3 Lymphocyte count decrease

Subject ID	AE Term	AE start/End dates	Dose	Dosing dates	Causality	Outcome		
**0030003**	Lymphocyte count decreased intermittently	11Oct 2017/	0.5 mg	10-14 Oct 2017	Possibly related	Ongoing		
	Screening 27/09/17	Day 1 10/10/17	Day 2 11/10/17	Day 3 12/10/17	Day 4 13/10/17	Day 5 14/10/17	Within 24 hrs 14/10/17	EOS 08/11/17
Leukocytes (10^9/L)	9.2	8.4	6.3	5.9	6.8	5.7	6	6.9
Neutrophils (10^9/L)	7.21	Not Done	5.09	5.3	5.71	4.39	5	Not Done
Lymphocyte count (10^9/L)	0.78 G2	Not Done	0.49 G3	0.3 G3	0.57 G2	0.77 G2	0.49 G3	0.41 G3
		Pre-infusion				End of Infusion		Week 4
BMP4 (pg/mL)		545				623		535
Withdrawal due to disease progression (08/11/17)- Death due to euthanasia (16/11/17)								
**Subject ID**	**AE Term**	**AE start/End dates**	**Dose**	**Dosing dates**	**Causality**	**Outcome**		
0030009	Lymphocyte count decreased	15Oct 2018/17 Oct 2018	9.0 mg	15-19 Oct 2018	Possibly related	Recovered		
	Screening 12/10/18	Day 1 15/10/18	Day 2 16/10/18	Day 3 17/10/18	Day 4 18/10/18	Day 5 19/10/18	Within 24 hrs 19/10/18	EOS 09/11/18
Leukocytes (10^9/L)	5.2	8.1	5.4	6.7	4.8	5.7	5.5	5.3
Neutrophils (10^9/L)	4.14	7.33	4.38	5.36	3.5	4.36	4.12	4.12
Lymphocyte count (10^9/L)	0.53 G2	0.29 G3	0.44 G3	0.53 G2	0.57 G2	0.62 G2	0.75 G2	0.57 G2
		Pre-infusion				End of Infusion		Week 4
BMP4 (pg/mL)		448				1319		520
Withdrawal due to disease progression (09/11/18)- Death due to disease progression (15/10/19)								

BMP4 circulating levels (pg/mL) in Responders (A) and Non Responders (B) suggesting that tumor response is independent of the systemic levels of BMP4; (C) BMP4 Serum levels in the 2 subjects with Grade 3 Lymphocyte count decrease showing no direct correlation between the BMP4 levels and Lymphocyte count decrease

Supplementary Table S4: Overview of reported adverse events

	**Number of events**					
Event	0·5 mg (n=3)	1·5 mg (n=3)	4·0 mg (n=3)	9·0 mg (n=3)	18 mg (n=3)	Total (n=15)
AEs	19	13	20	17	28	97
Related	1	0	0	1	6	8
SAEs	3	3	4	3	4	17
Related	0	0	0	0	0	0
DLT	0	0	0	0	0	0

Supplementary Table S5: Summary of Treatment Emergent Adverse Events (TEAE) by System Organ Class (in ≥ 25% of patients) and Preferred Term

Overall	hrBMP4 0·5 mg (N=3)	hrBMP4 1·5 mg (N=3)	hrBMP4 4 mg (N=3)	hrBMP4 9 mg (N=3)	hrBMP4 18 mg (N=3)	Overall (N=15)
	E n	E n	E n	E n	E n	E n (%)
**Any TEAE**	19 3	13 3	20 3	17 3	28 3	97 15 (100·0%)
**Blood and lymphatic system disorders**	1 1	-	-	3 2	1 1	5 4 (26·7%)
Anaemia	1 1	-	-	1 1	1 1	3 3 (20·0%)
Leukopenia	-	-	-	1 1	-	1 1 (6·7%)
Lymphopenia	-	-	-	1 1	-	1 1 (6·7%)
**Gastrointestinal disorders**	2 1	-	1 1	1 1	4 3	8 6 (40·0%)
Gastritis	-	-	-	1 1	-	1 1 (6·7%)
Nausea	1 1	-	-	-	1 1	2 2 (13·3%)
Vomiting	1 1	-	1 1	-	3 3	5 5 (33·3%)
**General disorders and administration site conditions**	1 1	-	3 2	-	2 1	6 4 (26·7%)
Catheter site haemorrhage	-	-	1 1	-	-	1 1 (6·7%)
Euthanasia	1 1	-	1 1	-	-	2 2 (13·3%)
Pain	-	-	-	-	1 1	1 1 (6·7%)
Pyrexia	-	-	1 1	-	1 1	2 2 (13·3%)
**Injury, poisoning and procedural complications**	1 1	-	1 1	-	2 2	4 4 (26·7%)
Wound	1 1	-	-	-	-	1 1 (6·7%)
Wound complication	-	-	1 1	-	1 1	2 2 (13·3%)
Wound infection	-	-	-	-	1 1	1 1 (6·7%)
**Investigations**	5 2	2 1	6 2	2 1	5 2	20 8 (53·3%)
Alanine aminotransferase increased	1 1	-	-	-	1 1	2 2 (13·3%)
Aspartate aminotransferase increased	1 1	-	1 1	-	1 1	3 3 (20·0%)
Blood bicarbonate increased	-	-	1 1	-	1 1	2 2 (13·3%)
Blood bilirubin increased	-	-	1 1	-	-	1 1 (6·7%)
Blood creatine decreased	-	-	-	-	1 1	1 1 (6·7%)
Blood creatinine increased	1 1	-	-	-	-	1 1 (6·7%)
Blood lactate dehydrogenase increased	-	1 1	1 1	1 1	-	3 3 (20·0%)
Gamma-glutamyltransferase increased	1 1	-	1 1	-	-	2 2 (13·3%)
Lymphocyte count decreased	1 1	-	1 1	1 1	1 1	4 4 (26·7%)
Red blood cell count decreased	-	1 1	-	-	-	1 1 (6·7%)
**Metabolism and nutrition disorders**	-	2 1	2 2	1 1	4 2	9 6 (40·0%)
Hyperglycaemia	-	1 1	2 2	-	1 1	4 4 (26·7%)
Hypoalbuminaemia	-	-	-	-	1 1	1 1 (6·7%)
Hypokalaemia	-	-	-	1 1	-	1 1 (6·7%)
Hypophosphataemia	-	1 1	-	-	2 2	3 3 (20·0%)

Overall	hrBMP4 0·5 mg (N=3)		hrBMP4 1·5 mg (N=3)		hrBMP4 4 mg (N=3)		hrBMP4 9 mg (N=3)		hrBMP4 18 mg (N=3)		Overall (N=15)	
	E	n	E	n	E	n	E	n	E	n	E	n (%)
**Neoplasms benign, malignant and unspecified (incl cysts and polyps)**	2	2	3	3	3	3	2	2	3	2	13	12 (80·0%)
Neoplasm progression	2	2	3	3	3	3	2	2	3	2	13	12 (80·0%)
**Nervous system disorders**	7	2	5	2	4	2	4	2	3	2	23	10 (66·7%)
Aphasia	1	1	-		-		-		-		1	1 (6·7%)
Extensor plantar response	1	1	-		-		-		-		1	1 (6·7%)
Headache	1	1	2	2	2	2	4	2	2	2	11	9 (60·0%)
Hemiparesis	2	2	1	1	2	1	-		-		5	4 (26·7%)
Muscle spasticity	1	1	-		-		-		-		1	1 (6·7%)
Nervous system disorders	-		1	1	-		-		-		1	1 (6·7%)
Seizure	-		-		-		-		1	1	1	1 (6·7%)
Sensory loss	1	1	-		-		-		-		1	1 (6·7%)
Tremor	-		1	1	-		-		-		1	1 (6·7%)

Supplementary Table S6: Overview of Quality-of-Life reporting

	0·5 mg		1·5 mg		4·0 mg		9·0 mg		18·0 mg		Total	
EORTC QLQ-C30	Mean	n	Mean	n	Mean	n	Mean	n	Mean	n	Mean	n
**Functioning scales**												
Physical												
Hospitalization	71	3	62	3	87	3	84	3	89	3	79	15
Week 4	29	3	60	2	89	3	70	2	84	3	67	13
Week 8	57	2			80	1			100	1	73	4
Week 12	43	2							100	1	62	3
Week 52	93	1			53	1			100	1	82	3
Role												
Hospitalization	39	3	22	3	56	3	83	3	61	3	52	15
Week 4	22	3	50	2	56	3	58	2	78	3	53	13
Week 8	33	2			33	1			100	1	50	4
Week 12	25	2							100	1	50	3
Week 52	33	1			33	1			67	1	44	3
Emotional												
Hospitalization	72	3	83	2	78	3	83	3	69	3	77	14
Week 4	67	3	38	2	61	3	71	2	81	3	65	13
Week 8	96	2			75	1			100	1	92	4
Week 12	63	2							100	1	75	3
Week 52	50	1			67	1			100	1	72	3
Cognitive												
Hospitalization	61	3	44	3	83	3	72	3	67	3	66	15
Week 4	50	3	33	2	61	3	67	2	67	3	56	13
Week 8	50	2			100	1			83	1	71	4
Week 12	33	2							83	1	50	3
Week 52	67	1			100	1			83	1	83	3
Social												
Hospitalization	56	3	50	3	94	3	78	3	89	3	73	15
Week 4	56	3	33	2	78	3	58	2	94	3	67	13
Week 8	33	2			83	1			100	1	63	4
Week 12	33	2							100	1	56	3
Week 52	50	1			83	1			83	1	72	3
Global												
Hospitalization	50	3	69	3	75	3	72	3	50	3	63	15
Week 4	36	3	58	2	61	3	58	2	67	3	56	13
Week 8	54	2			33	1			62	1	58	4
Week 12	50	2							83	1	61	3
Week 52	83	1			33	1			83	1	67	3

**Symptom scales**												
Fatigue												
Hospitalization	37	3	61	2	41	3	26	3	52	3	42	14
Week 4	63	3	72	2	37	3	50	2	30	3	49	13
Week 8	56	2			22	1			11	1	36	4
Week 12	50	2							0	1	33	3
Week 52	22	1			56	1			0	1	26	3
Nausea/vomiting												
Hospitalization	6	3	11	3	0	3	0	3	56	3	14	15
Week 4	6	3	8	2	11	3	0	2	0	3	5	13
Week 8	0	2			0	1			0	1	0	4
Week 12	0	2							0	1	0	3
Week 52	0	1			0	1			0	1	0	3
Pain												
Hospitalization	22	3	39	3	28	3	6	3	22	3	23	15
Week 4	33	3	25	2	28	3	8	2	11	3	22	13
Week 8	0	2			0	1			0	1	0	4
Week 12	0	2							0	1	0	3
Week 52	0	1			0	1			0	1	0	3
Dyspnea												
Hospitalization	22	3	44	3	0	3	0	3	11	3	16	15
Week 4	22	3	0	2	0	3	33	2	0	3	10	13
Week 8	0	2			0	1			0	1	0	4
Week 12	0	2							0	1	0	3
Week 52	0	1			0	1			0	1	0	3
Insomnia												
Hospitalization	44	3	78	3	22	3	11	3	56	3	42	15
Week 4	11	3	50	2	22	3	17	2	11	3	21	13
Week 8	0	2			0	1			0	1	0	4
Week 12	0	2							0	1	0	3
Week 52	33	1			0	1			0	1	11	3
Appetite loss												
Hospitalization	11	3	0	3	0	3	0	3	44	3	11	15
Week 4	0	3	17	2	0	3	50	2	0	3	10	13
Week 8	17	2			0	1			0	1	8	4
Week 12	0	2							0	1	0	3
Week 52	0	1			0	1			0	1	0	3
Constipation												
Hospitalization	11	3	33	3	11	3	11	3	44	3	22	15
Week 4	11	3	0	2	11	3	0	2	0	3	5	13
Week 8	17	2			0	1			0	1	8	4
Week 12	17	2							0	1	11	3
Week 52	0	1			0	1			0	1	0	3

Diarrhea												
Hospitalization	0	3	33	3	0	3	0	3	0	3	7	15
Week 4	0	3	0	2	0	3	0	2	0	3	0	13
Week 8	0	2			0	1			0	1	0	4
Week 12	0	2							0	1	0	3
Week 52	0	1			0	1			0	1	0	3
Financial difficulties												
Hospitalization	11	3	33	3	22	3	0	3	22	3	18	15
Week 4	11	3	50	2	11	3	0	2	0	3	13	13
Week 8	17	2			67	1			33	1	33	4
Week 12	17	2							33	1	22	3
Week 52	0	1			67	1			67	1	44	3

Supplementary Table S7: List of primers used for Sanger sequencing

Gene	**Mutation**	**Primer Forward**	**Primer Reverse**
ATRX	C.5375_5395del	TTTCAAAACGATATTCCAAGAG	ACTCCTATAATCATAATACATG
BRAF	c.G1465A	ACATTACTTGCAGTTTCAGTTAGT	GCTTGCTTCCCTGGTCTGCTCTCT
CDKN2A	c.G187C	AGCAGCATGGAGCCTTCGGCTGA	CAGCATTCGAGAGATCTGTACG
EGFR	c.C866T	CAGTAACTTGGGCTTTCTGA	AAGATGGGATACTCCAGGGC
EGFR	c.G1793T	GGTGCAATCACAGAATAACTGG	GTTGCCGGAAAACTTGGGAG
ERBB2	c.C3647A	AGCACGCAAGCTTTCTCCTGCTGT	CAGACATGACCTCGGCCAGCC
IDH1	c.G395A	ACCAAATGGCACCATACGA	TTCATACCTTGCTTAATGGGTGT
MET	c.C2962T	GTGCTACAACCTGTGTAGTAC	GAAATGAGAGCTTATGGACTC
NF1	c.C1591T	CGTCCAGCCTAGTTCTAGAACAT	GCAAGTAAACCCCTTCTTTCTC
PIK3CA	c.G1633A	ATCCAGAGGGGAAAAATATG	ATGCTGAGATCAGCCAAAT
PTEN	c.T593C	GTCAGAGCGCTGTTGTGACC	TATAATTTGGCTTCGACTAC
PTEN	c.955dupA	CTCAGATTGCCTTATAATAGT	CAAGTTCTTCATCAGCTGTACTC
PTEN	c.956_959del	CTCAGATTGCCTTATAATAGT	CAAGTTCTTCATCAGCTGTACTC
TP53	c.G830T	CCCCTGCTTGCCACAGGT	GTGAATCTGAGGCATAACTGC
TP53	c.A745G	CCCCTGCTTGCCACAGGT	GTGAATCTGAGGCATAACTGC
TP53	c.626_627del	GCCGTCTTCCAGTTGCTTTATCTG	TGGTGGTACAGTCAGAGCCAAC
TP53	c.G322A	CCTGGTCCTCTGACTGCTCTTTTCACCCA	GGCCAGGCATTGAAGTCTCAT
